# Metabolomic profiling of *Burkholderia cenocepacia* in synthetic cystic fibrosis sputum medium reveals nutrient environment-specific production of virulence factors

**DOI:** 10.1038/s41598-021-00421-4

**Published:** 2021-11-01

**Authors:** Olakunle A. Jaiyesimi, Andrew C. McAvoy, David N. Fogg, Neha Garg

**Affiliations:** 1 School of Chemistry and Biochemistry, Georgia Institute of Technology, 950 Atlantic Drive, Atlanta, GA 30332-2000 USA; 2 Center for Microbial Dynamics and Infection, Georgia Institute of Technology, 311 Ferst Drive, ES&T, Atlanta, GA 30332 USA

**Keywords:** Microbiology, Bacteria, Bacterial techniques and applications

## Abstract

Infections by *Burkholderia cenocepacia* lead to life-threatening disease in immunocompromised individuals, including those living with cystic fibrosis (CF). While genetic variation in various *B. cenocepacia* strains has been reported, it remains unclear how the chemical environment of CF lung influences the production of small molecule virulence factors by these strains. Here we compare metabolomes of three clinical *B. cenocepacia* strains in synthetic CF sputum medium (SCFM2) and in a routine laboratory medium (LB), in the presence and absence of the antibiotic trimethoprim. Using a mass spectrometry-based untargeted metabolomics approach, we identify several compound classes which are differentially produced in SCFM2 compared to LB media, including siderophores, antimicrobials, quorum sensing signals, and various lipids. Furthermore, we describe that specific metabolites are induced in the presence of the antibiotic trimethoprim only in SCFM2 when compared to LB. Herein, C13-acyl-homoserine lactone, a quorum sensing signal previously not known to be produced by *B. cenocepacia* as well as pyochelin-type siderophores were exclusively detected during growth in SCFM2 in the presence of trimethoprim. The comparative metabolomics approach described in this study provides insight into environment-dependent production of secondary metabolites by *B. cenocepacia* strains and suggests future work which could identify personalized strain-specific regulatory mechanisms involved in production of secondary metabolites. Investigations into whether antibiotics with different mechanisms of action induce similar metabolic alterations will inform development of combination treatments aimed at effective clearance of *Burkholderia* spp. pathogens.

## Introduction

Cystic fibrosis (CF) patients experience chronic lung infections by pathogenic microorganisms with accompanying airway inflammation, leading to a progressive decline in lung function and respiratory failure^1,2^. The *Burkholderia cepacia* complex (Bcc) is a group of over 20 related *Burkholderia* species which can cause chronic infections in CF patients^3,4^. Bcc bacteria display intrinsic and acquired resistance to many common antibiotics^5^. Additionally, Bcc spp. are capable of spreading between patients and can lead to cepacia syndrome, a condition characterized by rapidly progressing necrotizing pneumonia, bacteremia, and a sharp decline in lung function^5–9^. The majority of Bcc infections are caused by either *Burkholderia cenocepacia* or *Burkholderia multivorans*^10^. Bcc infections in general are associated with negative clinical outcomes^11,12^. In particular, *B. cenocepacia* and *B. multivorans* are the most feared CF pathogens as infections by this species are correlated with reduced survival rates and an increased risk of developing cepacia syndrome compared to other Bcc species^13–15^.

*Burkholderia* spp. bacteria are known to produce a variety of small organic molecules also known as natural products, allowing these bacteria to adapt and survive in diverse ecological niches^9,16–18^. These natural products play important roles in swarming, biofilm formation, iron acquisition, combating competition, and quorum sensing (QS). As such, many of these natural products serve as potential virulence factors. Genomic approaches have been utilized extensively to compare the potential of various Bcc spp*.* to produce virulence factors^19,20^. However, pathogens display variable phenotypes when cultured in diverse nutritional environments^21,22^. While genomic analysis provides insight into the metabolic potential of these bacteria, it does not accurately represent their metabolic state under specific environmental conditions. On the other hand, metabolomics is a complementary technique that provides a global “snapshot” of an organism’s metabolic state under a given condition and therefore is a relevant representation of the organism’s molecular phenotype. Thus, comparative metabolomics is an attractive approach for monitoring metabolic responses to external stimuli.

Previous work has demonstrated that nutrient availability at infection sites is associated with phenotypic changes in CF pathogens, including increased virulence^23,24^. Along these lines, clinical *B. cenocepacia* isolates cultured in a minimal medium supplemented with 12.5% (w/v) CF sputum were shown to exhibit differential expression of genes involved in virulence processes, such as antimicrobial resistance, iron uptake, protection against reactive oxygen/nitrogen species, motility, and secretion^25^. Therefore, it is evident that nutrient composition is an important consideration for developing a clinically relevant in vitro model of CF sputum to be used in comparative metabolomic studies. To this end, an assortment of artificial sputum media formulations have been established for studying CF pathogens in vitro that overcome the challenges with the inherent heterogeneity associated with culturing in sputum from patients^22,23,26–31^. Among these, a chemically defined synthetic CF sputum medium (SCFM) was developed, and subsequently refined (SCFM2) by adding DNA, mucin, 1,2-dioleoyl-sn-glycero-3-phosphocholine (DOPC), and *N*-acetylglucosamine (GlcNAc) to approximate the average physical and chemical environment of CF sputum^31,32^. The sputum of CF patients is a complex mixture of amino acids, carbohydrates, mucins, lipids, and micronutrients which support pathogen growth in vivo^23,24,33–38^. A transposon sequencing (Tn-seq) approach was used to demonstrate that genes required for fitness in vitro are practically identical in CF sputum and in SCFM2 medium for the prominent CF pathogen, *Pseudomonas aeruginosa*^31^. Additionally, the transcriptomic profiles of *P. aeruginosa* in CF sputum samples were best represented by an SCFM2-based in vitro model compared to any other single laboratory model tested^39^. While no individual laboratory model completely captured gene expression profiles observed during *P. aeruginosa* CF infections, the combination of SCFM2 and murine lung infection model came closest^39^.

These findings support the validity of SCFM2 medium as a model for studying CF pathogens in a controlled laboratory setting. To better understand small molecule production under disease-relevant conditions, we set out to compare the metabolomes of three *B. cenocepacia* strains when cultured in SCFM2 medium versus a standard laboratory growth medium. In this study, we demonstrate that *B. cenocepacia* strains exhibit altered metabolomic profiles when cultured in SCFM2 medium compared to Luria broth (LB). Specifically, we observed that growth in SCFM2 medium upregulates production of pyochelin-type siderophores while downregulating production of ornibactin siderophores. We also report production of unique *N*-acyl-homoserine lactone (AHL) QS signals in SCFM2, with C13-AHLs congeners detected exclusively in SCFM2 samples in the presence of trimethoprim. Additionally, we note that several families of lipids (including hopanoids, cyclopropane fatty acids, monoacylglycerols, and phosphatidylethanolamines) exhibit increased production in SCFM2 compared to LB. Finally, we show that sublethal concentrations of trimethoprim lead to media-dependent changes in secondary metabolite production, and that metabolism of trimethoprim itself is significantly upregulated during growth in SCFM2 compared to LB. Overall, this study provides valuable insight into *B. cenocepacia* secondary metabolite production under disease-relevant environmental conditions and highlights the importance of using appropriate in vitro models for metabolomic studies.

## Results and discussion

### Description of global changes in metabolome

**Figure 1:**
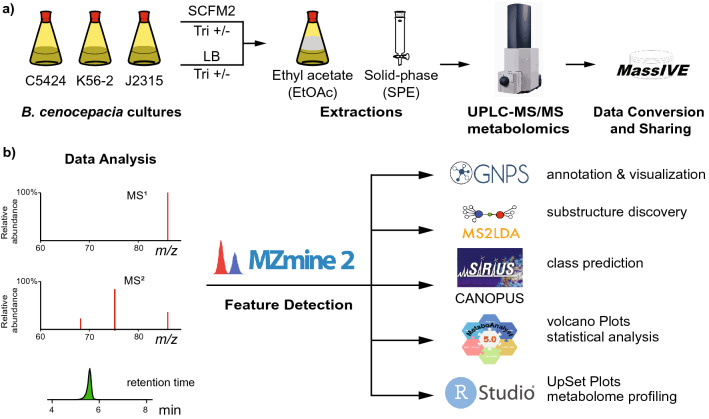
Summary of (**a**) experimental design and (**b**) data analysis workflow used in this study.

The nutritional composition of standard laboratory growth media such as LB is inadequate in modeling bacterial physiology during infection, as nutrient availability can vary markedly between infection sites and standard growth media. To overcome this challenge, the previously developed SCFM2 was used to model the physical and chemical environment of human sputum from patients with cystic fibrosis. In this study, we employed an untargeted metabolomic approach to compare the metabolomes of three different strains of *B. cenocepacia* namely, C5424, K56-2, and J2315 when cultured in SCFM2 and LB in triplicate (Fig. 1a). These strains are clonal and are associated with the highly transmissible, epidemic ET12 lineage of *B. cenocepacia*^19^. In addition, comparative metabolomic analysis was carried out in the presence and absence of trimethoprim, which is an antibiotic used clinically for treatment of *Burkholderia* infections^40^. This antibiotic is known to upregulate biosynthetic pathways involved in production of secondary metabolites in *B. thailandensis* and in the selected strains of *B. cenocepacia* in LB, but its effect has not been investigated in SCFM2^41–43^. In order to capture a diverse range of compound classes, extractions were performed on bacterial cultures using both liquid–liquid (with ethyl acetate, EtOAc) and solid-phase extraction (SPE) methods. Extracts were then analyzed using high resolution tandem mass spectrometry coupled with ultra-high-performance liquid chromatography (UHPLC-HRMS/MS). Metabolite features representing analytes detected at a unique *m/z* and retention time were extracted, aligned, and quantified using the open-source MZmine2 software and feature-based molecular networking was performed (Fig. 1b)^44,45^.

**Figure 2:**
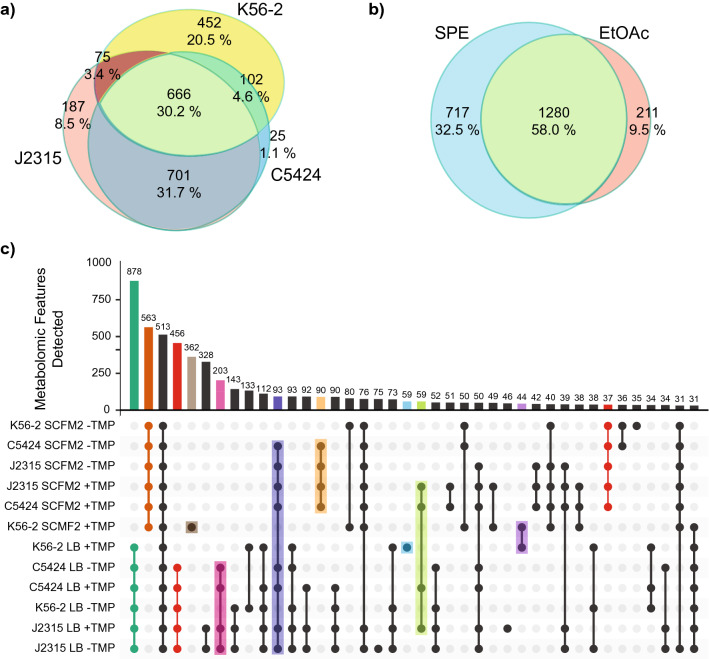
Overview of global metabolomic profiles across experimental conditions. (**a**) Euler Diagram showing distribution of metabolomic features across three *B. cenocepacia* strains used in this experiment, regardless of culturing conditions, time of harvest, or extraction methods used. (**b**) Euler diagram displaying features detected at 48 h with liquid–liquid extractions using EtOAc or solid-phase extraction methods (SPE), regardless of other experimental conditions. (**c**) UpSet plot displaying number of features detected in each combination of bacterial strain, growth media, and trimethoprim exposure condition. For the sake of clarity, only the top forty combinations of conditions in which the most unique features were detected are shown.

We first compared the metabolome of each strain irrespective of media type, extraction method, and exposure to antibiotic (Fig. 2a). The largest number of unique metabolite features (20.5%) was detected exclusively in the extracts of the strain K56-2, whereas 8.5% unique features were detected in the extracts of J2315 strain and 1.1% in the extracts of the strain C5424. While 30.2% of features were shared by all three strains, the largest number of features were shared between the strains J2315 and C54524 (31.7%). Among other factors, the similar metabotype of these two strains may be reflective of their ability to produce a brown pigment known as pyomelanin, which is not produced by the strain K56-2^46,47^. Next, we compared the metabolomes acquired using the SPE and EtOAc extraction methods separately at 48 h post-inoculum. This analysis revealed that more metabolite features were exclusively detected in extracts generated using SPE as compared to extracts generated with EtOAc (Fig. 2b). A total of 32.5% of the metabolite features were detected only with the SPE method, while 9.5% of the total features were unique to EtOAc extraction method (Fig. 2b). Thus, the extraction method employed results in biased metabolomics comparisons when only one type of extraction strategy is employed. Lastly, we generated an UpSet plot to visualize the number of features unique to each strain under different growth conditions as well as exposure to sub-lethal dose of antibiotic trimethoprim after subtraction of media background (Fig. 2c)^48^. This analysis revealed that media specific differences were the largest driver of metabolomic diversity within this study, with 878 features shared between all LB samples  and 563 features shared between all SCFM2 samples . In comparison, 513 features were detected in all samples. The UpSet plot also demonstrates that exposure to trimethoprim results in a unique metabolomic response by the K56-2 strain, with 362 metabolomic features exclusively detected in SCFM2 samples , 59 exclusive to LB samples , and 44 detected in both media types . Interestingly, 456 metabolomic features were uniquely detected in all LB samples except for K56-2 with trimethoprim  and 37 in all SCFM2 samples except for K56-2 with trimethoprim . While our prior studies have shown that K56-2 exhibits a unique metabolomic response to trimethoprim, our analysis demonstrates that this response is even more apparent in an environment representative of CF sputum^43^. Another pattern observed using UpSet plot highlighted that many features are uniquely detected as shared between the pigmented J2315 and C5424 strains, with 203 features uniquely detected in LB , 90 in SCFM2 , and 93 across both media types . An additional 59 features were uniquely detected in C5424 and J2315 strains cultured in LB and SCFM2 in the presence of trimethoprim , hinting at a distinct response to trimethoprim that is associated with pyomelanin production. Such responses are hallmark of the strain-specific phenotypes of *Burkholderia* observed while growing under conditions encountered during infection^49–52^. Future investigations into phenotypic differences as well as differences in gene expression via transcriptomics will provide insights into the biochemical underpinnings driving these observations. Specific metabolites underlying these personalized chemotypes are discussed below.

### Differences in siderophore production between LB and SCFM2 cultures

**Figure 3:**
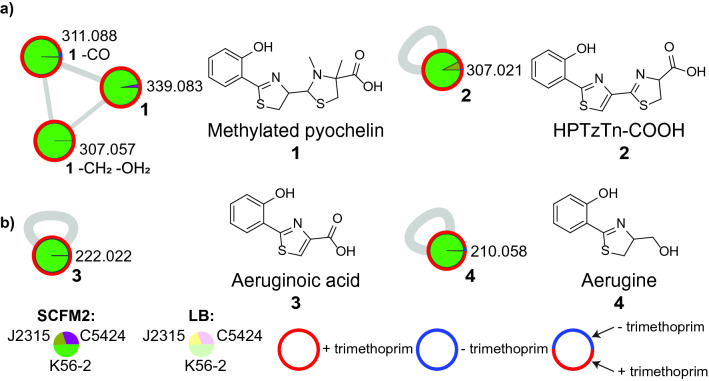
Analysis of pyochelin biosynthetic metabolite production across three *B. cenocepacia* strains. Pie charts within nodes correspond to relative abundance in each strain obtained via integration of area under the chromatographic peak (green: K56-2, purple: C5424, brown: J2315) and media condition (darker shade for SCFM2 and lighter shade for LB). The coloring of node border represents whether the metabolite was detected only in the presence of trimethoprim (red border), only in the absence of trimethoprim (blue), or both in the presence or absence of trimethoprim (mixed color). A combination of blue and red border color shows total relative abundance of the metabolite in the absence or presence of trimethoprim, respectively across all strains. The production of these compounds was only detected in SCFM2 medium, and in the presence of the antibiotic trimethoprim. (**a**) Annotated structural analogs of pyochelin, including methylated pyochelin (**1**) and HPTzTn-COOH (**2**). (**b**) Annotated shunt products of pyochelin biosynthesis, including aeruginoic acid (**3**) and aerugine (**4**).

Siderophores are compounds secreted by bacteria to acquire iron from the surrounding environment, and were among the metabolites which were differentially produced by *B. cenocepacia* strains in LB and SCFM2 in this study. In healthy mammalian hosts, the pool of free iron is limited due to poor solubility of iron in its ferric state under physiological conditions, and because the majority of iron is either located in intracellular compartments or bound by host proteins such as hemoglobin, transferrin, lactoferrin, and ferritin^53,54^. In contrast, levels of both free and ferritin-bound iron are higher in CF sputum compared to sputum from healthy hosts^55,56^. Since this cofactor is essential for many important biological processes, iron-acquisition is required for survival in the host environment and can influence microbe-microbe interactions^54,57^. Siderophores which are known to be produced by *Burkholderia* include pyochelin, ornibactins, salicylic acid, and cepabactin^43,54,58^. Among these, *B. cenocepacia* have been shown to produce ornibactin and pyochelin^54^. In our study, various structural analogs of pyochelin were detected exclusively in SCFM2 in the presence of trimethoprim (Fig. 3). Pyochelin itself was not detected in this study, despite prior reports of low-level production by *B. cenocepacia*. These prior reports did not use mass spectrometry or NMR to report pyochelin production, but relied on fluorescence-based thin layer chromatography^59^. Using MS^n^ analysis, we report production of a methylated derivative of pyochelin rather than pyochelin by *B. cenocepacia* strains used in this study as described below.

The feature with *m/z* 339.083 had a database annotation as a pyochelin methyl ester in GNPS (Supplementary Fig. S1a)^60^. However, pyochelin methyl ester has only been reported as a synthetic product generated to facilitate NMR characterization, and there is no available biosynthetic evidence that would support methylation on the carboxylic acid to produce an ester^61^. This feature was detected exclusively in cultures grown in SCFM2 supplemented with trimethoprim in both the K56-2 and C5424 strains, albeit with lower levels observed in C5424 (Fig. 3a). Comparisons with the GNPS library MS^2^ spectra of pyochelin revealed that the fragment peaks with *m/z* 120.045, 180.048, and 190.032 are shared between the two molecules while others were shifted by 14.015 Da (–CH_2_), supporting the annotation as a methylated analog of pyochelin (Supplementary Fig. S1b). A structure search of pyochelin in SciFinder revealed thiazostatin as a potential candidate. Thiazostatin A/B (**1**) are previously reported stereoisomeric natural products that are related to pyochelin by an additional C4″-methylation of the thiazolidine ring (Fig. 3a)^62,63^. This thiazolidine C4″-methylation has also been observed in structurally homologous metabolites including isopyochelin, watasemycin, and yersiniabactin^64–66^. By MS^2^ analysis alone, the location of the methylation cannot be unambiguously determined, although the mass shifts of the fragment ion with *m/z* 146.027 in the MS^2^ spectrum of pyochelin to *m/z* 160.043 in our unknown metabolite’s MS^2^ spectrum suggests it is found on the terminal thiazolidine ring. To confirm the position of methyl group, we conducted MS^3^ analysis on the fragment ion with *m/z* 186.058. This analysis revealed that fragmentation of 186.058 yields a MS^3^ ion with *m/z* 158.063, which indicates methylation on the thiazolidine ring rather than at the carboxylic acid (Supplementary Fig. S2). As this detected metabolite is likely produced by same biosynthetic gene cluster as pyochelin, it is likely that the stereochemistry is also the same. Thus, this feature is putatively annotated as enantiothiazostatin, labeled in Fig. 3 as methylated. Thiazostatin A/B have been found to display antioxidant activity, and further screening is necessary to determine if the detected compound possesses additional bioactivities^62^. Using MASST through the GNPS platform, we searched the MS^2^ spectrum of this compound against all public spectral datasets (Supplementary Fig. S3)^67^. This MASST search found dataset matches in extracts of *Pseudomonas* spp. grown in vitro, as well as in datasets analyzing the metabolomes of humans with various inflammatory diseases (including CF, diabetes, irritable bowel syndrome, rheumatoid arthritis, and HIV). Detection of this molecule in humans with inflammatory disease raises the possibility that this molecule may be important in influencing microbiome structure in the host, although further studies will be needed to explore whether this observation is truly associated with any biological significance.

Another metabolite with *m/z* 307.021 was detected exclusively in SCFM2 medium extracts in both the *B. cenocepacia* strains K56-2 and J2315. In our molecular network, this feature matched with 2′-(2-hydroxyphenyl)-4′-thiazolyl-2,4-thiazolinyl-4-carboxylic acid (HPTzTn-COOH, **2**) in the GNPS spectral library, which was further verified by manually comparing experimental MS^2^ spectra with a previously published MS^2^ spectra (Fig. 3a, Supplementary Fig. S4a)^68^. Like pyochelin, HPTzTn-COOH is a siderophore that is dependent on salicylic acid and cysteine as biosynthetic precursors and is capable of chelating Fe^3+^ in addition to other metal ions including Al^3+^, Ni^2+^, and Ca^2+^^68,69^. HPTzTn-COOH was detected exclusively in SCFM2 medium, and primarily in the K56-2 strain, although also at low levels in J2315 (Fig 3a). This metabolite exhibited increased production in the presence of trimethoprim for the K56-2 strain and was not detected in the absence of trimethoprim for the J2315 strain.

Next, a feature with *m/z* 222.022 was detected exclusively in K56-2 cultures grown in SCFM2 medium in the presence of trimethoprim (Fig. 3b, Supplementary Fig. S4b). This feature was annotated as aeruginoic acid (**3**), which is a shunt product in the pyochelin biosynthesis pathway observed in *Pseudomonas* and *Burkholderia* spp.^70,71^. Aeruginoic acid is the oxidized form of aeruginaldehyde (also known as the integrated quorum sensing signal, or IQS), which has been proposed as a “fourth QS molecule” in *P. aeruginosa*, although this claim has been disputed^72,73^. Due to the presence of the reactive aldehyde moiety in aeruginaldehyde, it reacts with complex natural products such as malleonitrone or mindapyrrole B^74,75^. Interestingly, pyochelin has been shown to spontaneously undergo cleavage and subsequent transformation into aeruginaldehyde when incubated in buffer solution at 30 °C^74^. Another shunt product of pyochelin biosynthesis with *m/z* 210.058 was detected under the same conditions as aeruginoic acid, and annotated as aerugine (**4**) (Fig. 3b, Supplementary Fig. S4c). Aerugine has been previously isolated from *Pseudomonas fluorescens* and exhibits selective antifungal activity^76^. Recent work by Kaplan et al. has indicated that aeruginaldehyde, aerugine, and aeruginoic acid have iron-binding activity with aeruginoic acid binding to Fe^3+^ with a 1:1 ratio, aeruginaldehyde binding with a 2:1 ratio, and aerugine binding with a 3:1 ratio^71^. Unlike pyochelin, aeruginoic acid has a specific affinity for iron compared to other biologically relevant metals^71^. The largest abundance of methylated pyochelin is observed in the strain K56-2, which is likely why these intermediates are also detected in the strain K56-2.

In contrast to metabolites from the pyochelin pathway described above, the ornibactin class of siderophores was detected in the culture extracts of LB medium and not detected in culture extracts of SCFM2 (Supplementary Fig. S5). The SCFM2 medium is prepared by adding 3.60 µM iron in the form of Fe_3_SO_4_, which was observed to be the average iron concentration present in expectorated sputum collected from CF patients^23^. Recently, it has been reported that commercial sources of mucin can be contaminating sources of iron that lead to altered siderophore production in *P. aeruginosa* cultured in SCFM2^22^. To determine whether the concentration of iron might account for the differential production of siderophores observed between SCFM2 and LB media, inductively coupled plasma mass spectrometry (ICP-MS) was performed on media aliquots. ICP-MS analysis revealed that SCFM2 contained a mean iron concentration of 5.25 µM (standard deviation of 0.52 µM) while LB contained a similar mean concentration of 4.73 µM (standard deviation of 0.62 µM) (Supplementary Fig. S6). Therefore, factors other than iron availability likely play a role in the expression of the pyochelin and ornibactin biosynthetic gene clusters^11,25^. QS systems have been previously implicated in regulating production of both pyochelin and ornibactin, with the CepR transcriptional regulator repressing production of ornibactin and the CepR2 regulator activating production of pyochelin in *B. cenocepacia* H111^77^. In our experiment, production of siderophores was observed to be dependent on strain and nutritional environment. The production of metabolites from the pyochelin pathway was further induced by the antibiotic trimethoprim, while ornibactins were not. Thus, a specific chemical cue in SCFM2 in presence of trimethoprim might play a role in induction of pyochelin production under these conditions and warrants detailed investigation in the future with knockout strains that lack QS circuitry. This observation is noteworthy as pyochelin production by *B. cepacia* was previously suggested to be correlated with morbidity and mortality in patients with CF^78^. Mechanistic investigations into selective induction of siderophore biosynthesis pathways are critical to understanding their relevance to infections, and as such require further inquiry.

### *N*-acyl-homoserine lactones production in LB compared to SCFM2

**Figure 4:**
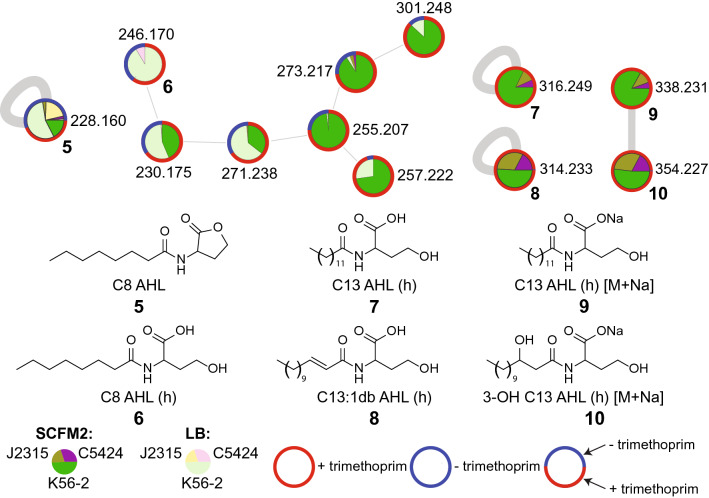
Distribution of *N*-acyl-homoserine lactones (AHL) and their hydrolyzed forms detected across three *B. cenocepacia* strains in both LB and SCFM2 media. Pie charts on nodes correspond to relative abundance in strain and media condition. The coloring of node border represents whether the metabolite was detected only in the presence of trimethoprim (red border), only in the absence of trimethoprim (blue), or both in the presence or absence of trimethoprim (mixed color). A combination of blue and red border color shows total relative abundance of the metabolite in the absence or presence of trimethoprim, respectively across all strains. The structures for C8-AHL (**5**), hydrolyzed C8-AHL (**6**), hydrolyzed C13-AHL (**7**), and hydrolyzed C13:1db-AHL (**8**) as well as sodium adducts of hydrolyzed C13-AHL (**9**) and hydrolyzed 3-OH-C13-AHL are shown (**10**).

QS mediates bacterial response to changing environmental conditions through cell-density dependent global changes in gene expression^79,80^. *Burkholderia* can utilize QS to coordinate various metabolic processes and modulate cellular phenotypes such as swarming, aggregation, spatial structuring, and biofilm formation^81,82^. QS also plays an important role in infection by regulating genes involved in virulence, and so establishing how the external environment influences production of QS signals is important to understanding their role in pathogenesis^83–85^. The CepIR and CciIR QS systems have been described in *B. cenocepacia* strains which primarily produce and sense *N*-octanoyl-homoserine lactone (C8-AHL, **5**) and *N*-hexanoyl-homoserine lactone (C6-AHL) respectively. In addition, an orphan LuxR-type regulator called CepR2, which is antagonized by C8-AHL, is also present in these strains^77,86–88^. The gene for the orphan CepR2 regulator lacks an adjacent gene required for synthesis for a cognate *N*-acyl-homoserine lactone. In a previous untargeted metabolomic experiment, we observed production of a wide diversity of AHLs by *Burkholderia* spp. grown in LB medium, as well as their corresponding acyl-homoserine products formed by hydrolysis of the lactone ring (referred to hereafter as “hydrolyzed AHLs”)^43^. In this study, we queried whether these signals are differentially detected in SCFM2 compared to LB media. The C8-AHL (*m/z* 228.160, **5**) was detected in both LB and SCFM2 media, and its hydrolyzed form (**6**) was detected in LB medium alone (Fig. 4). Additionally, we detected hydrolyzed C13-AHL (**7**), hydrolyzed C13-AHL:1db (**8**), the sodium adduct of hydrolyzed C13-AHL (**9**), and the sodium adduct of hydrolyzed 3-OH C13-AHL (**10**) exclusively in SCFM2 when trimethoprim was present (Fig. 4). Thus, the production of these C13-AHLs were induced by trimethoprim only in SCFM2. Detection of C8-AHL, hydrolyzed C8-AHL and hydrolyzed C13-AHL was confirmed using commercial AHL standards. These AHL standards were treated with sodium hydroxide to promote hydrolysis of the lactone ring to verify the detection of hydrolyzed AHLs (Supplementary Fig. S7). Naturally produced AHLs with an odd number of carbons in the acyl sidechain are relatively rare, and their functions are not well-characterized^89^. In a previous untargeted metabolomics study of 10 different *Burkholderia* strains grown in LB medium, we detected hydrolyzed 3-oxo-C13-AHL:1db exclusively in extracts of *B. thailandensis* E264^43^. To our knowledge, the present study represents the first description of C13 AHLs being produced by *B. cenocepacia*. Further studies will be needed to explore the function and biochemical basis for production of C13-AHLs by *B. cenocepacia* in SCFM2 medium, and identify the mechanism by which trimethoprim upregulates the production of this AHL.

### Fragin and pyrazine secondary metabolites

**Figure 5:**
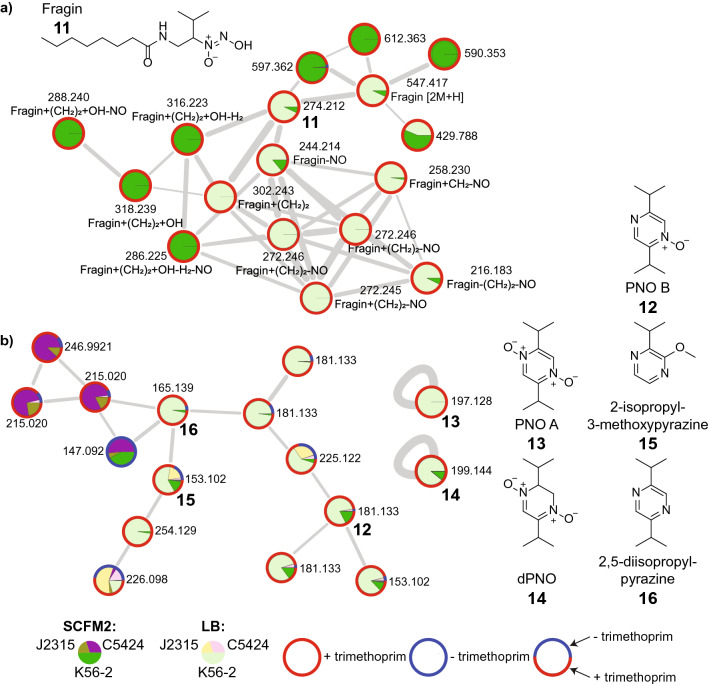
Distribution of fragin and pyrazine metabolites in SCFM2 and LB media. Pie charts on nodes correspond to relative abundance in strain and media condition. The coloring of node border represents whether the metabolite was detected only in the presence of trimethoprim (red border), only in the absence of trimethoprim (blue), or both in the presence or absence of trimethoprim (mixed color). A combination of blue and red border color shows total relative abundance of the metabolite in the absence or presence of trimethoprim, respectively across all strains. (**a**) Annotated fragin (**11**) cluster, which includes structural analogs and their in- source fragment ions which are characteristic of the diazeniumdiolate moiety. (**b**) Annotated nodes corresponding to pyrazines. The structures of pyrazine *N*-oxides PNO B (**12**), PNO A (**13**), dPNO (**14**), 2-isopropyl-3-methoxypyrazine (**15**) and 2,5-diisopropylpyrazine (**16**) are shown.

Fragin (**11**) is a diazeniumdiolate metallophore with antifungal activity that is produced by *Burkholderia* and *Pseudomonas* spp.^90^. In a previous untargeted metabolomics study, we reported significantly increased production of fragin and its structural analogs in *B. cenocepacia* K56-2 cultures grown in LB medium when supplemented with the antibiotic trimethoprim^43^. Fragin production was only observed in the K56-2 strain, despite the fact that the *ham* gene cluster responsible for fragin biosynthesis is identical in the closely related J2315 and C5424 strains. This result highlighted that even genetically similar *Burkholderia* strains can exhibit markedly different responses to external stimuli, such as trimethoprim exposure. In the current study, fragin was detected in both media conditions, but the majority of other nodes in the cluster (11/17) were detected exclusively in either LB or SCFM2 media (Fig. 5a). These analogs differ in the length of the acyl group added by the HamF enzyme, likely a result of differential availability of compounds containing variable acyl chain lengths which act as substrates for HamF. The MS^2^ spectra of these analogs showed a characteristic fragment corresponding to the loss of NO group (29.998 Da), that was also observed in the MS^1^ spectrum as an in-source fragment^43^. Fragin analogs with differential production in the two media types include nodes with *m/z* 302.243 (and the corresponding in-source fragment with *m/z* 272.246), 316.223 (in-source fragment with *m/z* 286.225), and 318.239 (in-source fragment with *m/z* 288.240) (Fig. 5a).

We discovered another cluster in our molecular network containing several molecules which, like fragin, are exclusively detected in K56-2 samples and show increased production in the presence of trimethoprim (Fig. 5b). These metabolites were reported by our group in a previous study as unknown metabolites^43^. Here we employed the recently developed CANOPUS tool to classify these unknown compounds into ClassyFire chemical classes, leading to their annotation as pyrazines^91,92^. This information led us to conduct a literature search of pyrazines produced by *Burkholderia*, enabling annotation of these metabolites as pyrazine *N*-oxides (PNOs), which was verified using standards provided by Li and colleagues (Supplementary Figs. S8, S9)^93^. The expression of *pvfB* and *pvfC* genes in animal pathogen *Pseudomonas entomophila* L48 and the plant pathogen *Pseudomonas syringae* pv. *syringae* UMAF0158 were previously shown to lead to production of a family of PNOs, namely PNO B (**12**, *m/z* 181.135), PNO A (**13**, *m/z* 197.126), and dPNO (**14**, *m/z* 199.145)^93^. The *pvfB* and *pvfC* genes are homologous to the *Burkholderia* genes *hamC* and *hamD* present in the biosynthetic gene cluster of fragin. Disruption of *pvfA-D* cluster in animal pathogen *Pseudomonas entomophila* L48 and the plant pathogen *Pseudomonas syringae* pv. *syringae* UMAF0158 was shown to significantly reduce the virulence of these strains^93^. Subsequent studies found that the *pvf* gene cluster is involved in synthesis of a signaling molecule which regulates production of small molecule virulence factors such as monalysin in *P. entomophilia* and mangotoxin in *P. syringae*^94–96^. Both dPNO and PNO B appeared in the feature-based molecular network, while PNO A did not due to low abundance. However, a node corresponding to PNO A was observed when data was analyzed with a classical molecular network (Fig. 5b). Two additional nodes in this network were putatively annotated as 2-isopropyl-3- methoxypyrazine (*m/z* 153.102, **15**) and 2,5-diisopropylpyrazine (*m/z* 165.138, **16**) based upon available literature of bacterially produced pyrazines (Fig. 5b, Supplementary Fig. S9)^97^. The MS^2^ spectra of these related molecules did not have fragment ions in common, and so molecular networking methods alone failed to highlight the structural relatedness of these three compounds. Nevertheless, CANOPUS enabled us to independently predict that each of these compounds were pyrazines, ultimately leading to their annotation.

These observations reveal that the *ham* gene cluster that is responsible for fragin biosynthesis also leads to production of PNOs in *B. cenocepacia*. PNO production was also induced by the addition of trimethoprim in both the SCFM2 and LB culture media. Thus, unlike the siderophores described above which showed media-dependent induction by trimethoprim, fragin and PNOs are similarly produced in the presence of antibiotic trimethoprim in both LB and SCFM2. The production of fragin and pyrazines including PNOs in the presence of trimethoprim in both SCFM2 and LB media highlights that antibiotics can serve as signaling molecules capable of significantly modulating expression of the genes encoding virulence factors across multiple nutritional environments.

### Differential detection of lipids in LB and SCFM2

**Figure 6:**
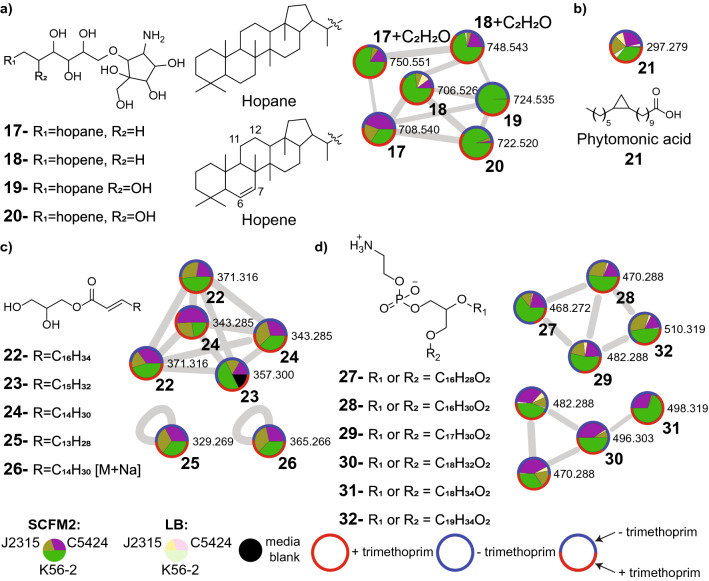
Distribution of various lipids detected across three *B. cenocepacia* strains in both LB and SCFM2 media. Pie charts within nodes correspond to relative abundance in each strain (green: K56-2, purple: C5424, brown: J2315) and media condition (darker shade for SCFM2 and lighter shade for LB). The coloring of node border represents whether the metabolite was detected only in the presence of trimethoprim (red border), only in the absence of trimethoprim (blue), or both in the presence or absence of trimethoprim (mixed color). A combination of blue and red border color shows total relative abundance of the metabolite in the absence or presence of trimethoprim, respectively across all strains. (**a**) Annotated hopanoid cluster, including bacteriohopanetetrol (BHT) cyclitol ether (**17**), bacteriohop-6-enetetrol cyclitol ether (**18**), bacteriohopanepentol (BHP) cyclitol ether (**19**), and bacteriohop-6-enepentol cyclitol ether (**20**). Additionally, an acetylated analog of BHT cyclitol ether (*m/z* 750.551) and an acetylated analog of bacteriohop-6-enetetrol cyclitol ether (*m/z* 748.543) were detected. (**b**) Node corresponding to phytomonic acid (**21**). (**c**) Annotated cluster and nodes representing monoacylglycerols (MGs). Detected MGs vary by number of carbon units on fatty acid chain, and include MG(19:1) (**22**), MG(18:1) (**23**), MG(17:1) (**24**), MG(16:1) (**25**), and the [M + Na] adduct of MG(17:1) (**26**). (**d**) Clusters corresponding to phosphatidylethanolamines (PEs). The PEs varied by acyl chain length, and were annotated as 2-OH-PE(16:1) (**27**), 2-OH-PE(16:0) (**28**), 2-OH-PE(17:1) (**29**), 2-OH-PE(18:1) (**30**), 2-OH-PE(18:0) (**31**) and 2-OH-PE(19:1) (**32**).

The overall lipid composition of bacterial cells has been reported to be influenced by several local environmental factors, including pH, nutrient availability, oxygen levels, temperature, and buildup of metabolic waste products^98^. Several clusters with hits to lipids in the GNPS spectral database were detected at higher levels in bacterial extracts grown in SCFM2, including hopanoids, phytomonic acid, monosaturated monoacylglycerols (MGs), and phosphatidylethanolamines (PEs), described below (Fig. 6).

Annotation of the hopanoid cluster was performed by first searching for candidate features with *m/z* calculated for known bacterial hopanoids. Identified candidate features were then verified by comparing experimental MS^2^ spectra to spectra previously published in literature^99,100^. Once annotations were supported through spectral matching, these features were used to further propagate annotations to connected nodes within the molecular network based on mass differences. Hopanoids are pentacyclic triterpenoids frequently found in bacterial membranes^101,102^. These polycyclic lipids are structurally analogous to eukaryotic sterols and are thought to have similar functions in regulating fluidity, permeability, and stabilization of bacterial membranes. Hopanoid biosynthesis has been previously reported in *B. cenocepacia*, where they are involved in resistance to low pH and antibiotics while also being important for swimming and swarming motility^101,102^. A metabolite feature with *m/z* 708.540 is annotated as bacteriohopanetetrol (BHT) cyclitol ether (**17**) and was detected only in extracts of SCFM2 (Fig. 6a, Supplementary Fig. S10a)^99,102^. Production of BHT cyclitol ether has been previously reported in *B. cenocepacia*, and our predicted annotation was supported by comparing experimental MS^2^ spectra with previously published spectra available in literature^99,102^. In addition, we annotated the node with *m/z* 706.526 as unsaturated BHT cyclitol ether (**18**) (Fig. 6a). Both Δ^6^ and Δ^11^ monosaturated BHT analogs have been previously characterized in bacteria^100^. Although our mass spectrometry analysis alone is insufficient to pinpoint the location of this unsaturation, we have putatively annotated this feature as bacteriohop-6-enetetrol cyclitol ether (**18**) since unsaturation at this location has been reported in *B. cepacia* strains^102,103^. This feature was detected in all three *B. cenocepacia* strains when grown in SCFM2 medium with the highest intensity observed in the K56-2 strain, but when cultured in LB it was only detected in J2315 cultures at low levels. Annotation of another feature with *m/z* 724.535 is consistent with a gain of a hydroxyl group from BHT cyclitol ether (**17**). This feature was exclusively detected in K56-2 strains grown in SCFM2 medium and the associated MS^2^ spectra indicates this feature is likely bacteriohopanepentol (BHP) cyclitol ether (**19**) (Supplementary Fig. S10b). BHP derivatives have been fully characterized only in *Acetobacter* spp., *Azotobacter vinelandii*, and *Nostoc* spp., although our observation is supported by prior evidence suggesting BHP derivatives are produced by *B. cepacia* strains as well^103–106^. The feature with *m/z* 722.520 was detected in all three *B. cenocepacia* strains (largely in K56-2 samples) grown in SCFM2 medium, which is presumably bacteriohop-6-enepentol cyclitol ether (**20**) although the location of unsaturation cannot be unambiguously determined as mentioned above. Next, a feature with *m/z* 750.551 was detected exclusively in SCFM2 containing cultures with a mass difference of 42.011 (C_2_H_2_O) from BHT cyclitol ether representative of acetylation. The final feature in the hopanoid cluster with *m/z* of 748.543 is annotated as unsaturated analog of the acetylated BHT cyclitol ether (*m/z* 750.551), with the unsaturation most likely occurring at the C6 position.

Another metabolite in the lipid family is annotated as phytomonic acid (**21**) based on the spectral match to the MS^2^ spectrum in the GNPS database. This annotation was confirmed using a commercial analytical standard (Fig. 6b, Supplementary Fig. S11a). Phytomonic acid was detected in both LB and SCFM2 media, although consistently higher levels were detected in SCFM2 medium for all three strains (Supplementary Fig. S11b). Production of cyclopropane acids have been previously reported in *B. multivorans*^107^. Cyclopropane fatty acids such as phytomonic acid are suggested to regulate membrane fluidity and stability and have been shown to increase extracellular survival in acidic or under conditions of high osmolarity^108,109^. These lipids also are major components of the membranes of intracellular pathogens such as *Brucella abortus* and *Mycobacterium tuberculosis*^108,109^. It is interesting to note that similar to *B. abortus* and *M. tuberculosis*, *Burkholderia* spp. are also capable of intracellularly infecting macrophage cells. The specific role of cyclopropane fatty acids in *B. cenocepacia* virulence requires further investigation.

Multiple monounsaturated monoacylglycerol (MG) lipids including nonadecenoyl-glycerol (19:1 MG, **22**), octadecenoyl-glycerol (18:1 MG, **23**), heptadecenoyl-glycerol (17:1 MG, **24**), hexadecanoyl-glycerol (16:1 MG, **25**), and the sodium adduct of heptadecenoyl-glycerol (17:1 MG, **26**) were detected only during growth in SCFM2. Annotations for this class of lipids was performed with a commercial standard of octadecenoyl(d7)-glycerol (18:1-d(7) MG) (Fig. 6c, Supplementary Fig. S12). For both 19:1 MG (**22**) and 17:1 MG (**24**), two unique metabolic features were detected with identical masses and MS^2^ patterns but slightly different retention times, possibly corresponding to distinct *cis/trans* isomers. *Burkholderia* spp. are known to produce the lipase LipA, which yields monoacylglycerols as a product during degradation of di- and tri-acylglycerides^110,111^. In *B. cenocepacia*, production of the LipA lipase is induced as part of the CepIR quorum sensing system^86,112^. Due to the ability of monoacylglycerols to destabilize bacterial cell membranes, several have been reported to demonstrate antimicrobial activities which vary based on chain length and degree of unsaturation^113,114^.

Finally, several phosphatidylethanolamines (PEs) were detected in this study, all consistently detected under similar conditions. In Gram-negative bacteria, the inner leaflet of the outer membrane is made up of phospholipids, of which PEs are the major component^115^. These PEs varied by the length of their acyl-chains, and were annotated as 2-OH-PE(16:1) (**27**), 2-OH-PE(16:0) (**28**), 2-OH-PE(17:1) (**29**), 2-OH-PE(18:1) (**30**), 2-OH-PE(18:0) (**31**), and 2-OH-PE(19:1) (**32**) based on GNPS library matching and manual annotations based on characteristic MS^2^ fragmentation patterns described in literature (Fig. 6d)^116^. Production of 2-OH-PEs has been observed in *B. cepacia*, and is reported to increase during stress observed under growth in high temperature^117^.

### Differential metabolism of the antibiotic trimethoprim between LB and SCFM2

**Figure 7:**
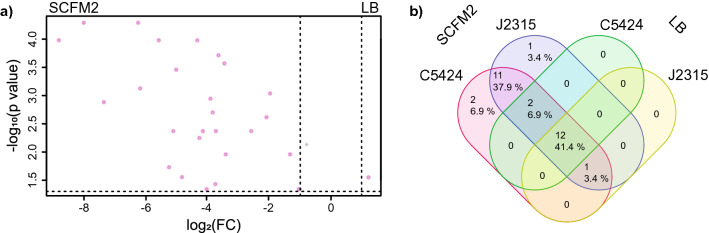
Distribution of metabolites containing trimethoprim motif which were produced by pigmented *B. cenocepacia* strains (C5424 and J2315) in SCFM2 and LB media. (**a**) Volcano plot showing trimethoprim metabolites which are differentially detected when cultured in either LB (right) or SCFM2 (left). Metabolites exhibiting an absolute fold change greater than 2 and a p value less than 0.05 (indicated by pink dots) were considered differentially detected. (**b**) Venn diagram showing the distribution of trimethoprim metabolites in C5424 and J2315, cultured in SCFM2 and LB media.

Several compounds that were differentially detected between growth in SCFM2 and LB were found to be related to trimethoprim as evidenced by MS^2^ spectral similarity. As previously described, MS2LDA was used to discover metabolites that contained trimethoprim as substructure. Thus, being higher in molecular weight than trimethoprim, these metabolites represent biochemical transformations of trimethoprim itself by *B. cenocepacia* bacteria^43^. In this study, MS2LDA Mass2Motif 543 was annotated as a trimethoprim substructure. We compared the presence of these metabolites in the extracts of bacteria cultured in LB and SCFM2 (Fig. 7). Metabolism of trimethoprim was carried out by only the pigmented strains J2315 and C5424 and not by the non-pigmented K56-2 strain, as reported previously^43^. We generated a volcano plot of molecules containing the trimethoprim motif to visualize compounds which were differentially detected across the two media conditions (Fig. 7a). This analysis revealed that the majority of the trimethoprim metabolites showed significantly higher production in SCFM2 as compared to LB. Structural characterization of these metabolites will provide insight into the pathways used by pigmented Bcc strains to metabolize xenobiotic compounds like the antibiotic trimethoprim and will facilitate future studies exploring how biotransformation will impact antibacterial activity.

## Conclusion

It is well established that environmental conditions are major drivers of secondary metabolite production in bacteria. Therefore, selecting an appropriate culture media for in vitro bacterial growth is crucial for designing metabolomic studies relevant to the biological system of interest. In this study, we characterized the metabolomic profiles of three clinical *B. cenocepacia* isolates in SCFM2 and LB media. Sublethal concentrations of trimethoprim has previously been shown to induce secondary metabolite production in *Burkholderia* spp. Thus, culturing was also performed with and without this antibiotic to understand how trimethoprim-induced metabolomic responses vary under CF relevant environmental conditions^42,43^. We demonstrate considerable metabolic variability between SCFM2 and LB media. In particular, we report that growth in SCFM2 medium upregulates production of pyochelin-type siderophores in the presence of trimethoprim while downregulating production of ornibactin siderophores compared to LB medium. We also note that AHL quorum sensing signals are differentially produced between the two media, with C13-AHLs exclusively detected in SCFM2 in the presence of antibiotic trimethoprim. Thus, trimethoprim induces both the production of pyochelin-type siderophores and a specific AHL signal in SCFM2 medium and not in LB. Moreover, we observed that metabolism of trimethoprim itself is significantly upregulated in SCFM2 medium compared to LB. Finally, we show that several lipid families (including hopanoids, cyclopropane fatty acids, monoacylglycerols, and phosphatidylethanolamines) exhibit increased production in SCFM2 compared to LB. While further work is needed to fully understand the biochemical mechanisms underlying these findings, this study provides insight into variable production of secondary metabolite production by *Burkholderia cenocepacia* spp. and is important for delineating personalized metabotypes of different strains of the same species in an in vitro model of CF. Even though metabolomics approaches have been significantly advanced in the last decade enabling detection of low-abundance metabolites, it remains a challenge to directly detect and identify a diversity of pathogen-specific chemical signals in clinical samples such as human sputum since host biomass typically vastly exceeds microbial biomass. Thus, interrogation of pathogenic strains isolated from infection sites in simplified model systems capable of inducing chemical signals observed during infection serves as an excellent discovery tool. Characterizing metabolomic profiles of *B. cenocepacia* strains isolated from infection sites and cultured in SCFM2 medium in the presence of antibiotics can become a valuable approach to guide appropriate use of combination treatments for effective pathogen clearance.

## Methods

### Bacterial strains

*Burkholderia cenocepacia* C5424, K56-2, and J2315 clinical isolates were used for culturing during this study^59,118,119^. These strains belong to the highly transmissible and epidemic ET12 lineage, wherein the J2315 and K56-2 strains are clonally related. As reported, the C5424 and J2315 strains produced observable amounts of the pigment pyomelanin whereas the K56-2 strain did not^46^.

### Media formulation and growth conditions

SCFM2 medium was prepared as previously described by Turner and co-workers^31^. All culturing was performed following previously established methods^43^. Briefly, *B. cenocepacia* C5424, J2315, and K56-2 frozen glycerol stocks were streaked onto LB agar plates, and then incubated overnight at 37 °C. Subsequently, 5 mL of LB media was inoculated using colonies from the overnight incubated plates, which was then incubated overnight at 37 °C while shaking at 250 rpm. After 16 h, sterile 125 mL Erlenmeyer flasks containing 20 mL of either LB-50 mM MOPS (pH 7.0) or SCFM2 media were inoculated with each overnight culture at an initial OD_600_ of 0.05. For each strain and media combination, four flasks were supplemented with 50 μL of a 12 mM trimethoprim stock (suspended in DMSO) to a final concentration of 30 μM, and four flasks were supplemented with 50 μL of DMSO as a vehicle control. These cultures were grown at 37 °C while shaking at 250 rpm. Half of the culture (10 mL) from each combination of strain and media type was harvested after 24 h (four containing trimethoprim and four with DMSO instead of trimethoprim), and the remaining half (10 mL) was harvested at 48 h.

### Sample extraction

Features were extracted using our previously established methods^43^. Briefly, each culture was extracted using two different methods: liquid–liquid extraction with ethyl acetate (EtOAc) and solid-phase extraction (SPE) using C18 columns (100 mg bed weight). For EtOAc extractions, 20 mL of the solvent was added to whole cultures and mixed every 30 min for 2 h before centrifuging at 2000×*g* for 3 min. The organic layer (top) was aspirated off with a glass pipette and transferred to a scintillation vial before drying in vacuo with a centrifugal evaporator. For solid-phase extractions, cultures were centrifuged at 10,000×*g* for 10 min and decanted to remove supernatant fluid. SPE columns were initially washed with 10 mL of 100% MeCN, subsequently equilibrated with water (10 mL) and then loaded with the culture supernatant. Analytes were then sequentially eluted from the column using 5 mL each of 20%, 50% and 100% MeCN. These three fractions were pooled together and dried in vacuo with a centrifugal evaporator.

### Ultra-high-performance liquid chromatography tandem mass spectrometry (UHPLC-MS/MS) data acquisition

Dried extracts were dissolved in 600 µL of 80% MeOH (LC–MS grade) containing 1 µM sulfadimethoxine as an internal standard before analyzing on an Agilent 1290 Infinity II UHPLC system (Agilent Technologies) coupled to an ImpactII ultra-high resolution Qq-TOF mass spectrometer (Bruker Daltonics, GmbH, Bremen, Germany) equipped with ESI source. Chromatographic separation was achieved on a KinetexTM 1.7 µm C18 reversed phase UHPLC column (50 × 2.1 mm). Solvent A consisted of water with 0.1% (v/v) formic acid and solvent B consisted of MeCN with 0.1% (v/v) formic acid. The gradient used for chromatographic separation consisted of 5% B and 95% A for 2 min, a linear increase to 95% B over 17 min, a hold for 3 min, a linear decrease to 5% B in 1 min, and hold for 1 min with a constant flow rate of 0.5 mL/min throughout. For MS spectra acquisition, the instrument was set to capture features from *m/z* 50–2000 Da in positive ion mode. External calibration was performed prior to data collection using ESI-L Low Concentration Tuning Mix (Agilent Technologies), with hexakis(1H,1H,2H-perfluoroethoxy)phosphazene employed as internal lock-mass calibrant throughout the run. Ion source parameters were set to 4500 V for capillary voltage, 2 bar for nebulizer gas pressure (N_2_), 200 °C for ion source temperature, 9 L/min for dry gas flow, and a spectral rate of Hz for MS^1^ and 6 Hz for MS^2^. For MS^2^ data acquisition, the eight most intense precursor ions per MS^1^ scan were selected for MS^2^ fragmentation. A basic stepping function was used to fragment ions at 50% and 125% of the CID calculated for each *m/z*, with timing of 50% for each step. Similarly, a basic stepping of collision RF of 550 and 800 Vpp with a timing of 50% for each step and transfer time stepping of 75 and 90 µs with a timing of 50% for each step was employed. The MS/MS active exclusion parameter was set to 2 and the active exclusion was released after 30 s. The mass of the internal lock-mass calibrant was excluded from MS^2^ acquisition. A mixture of 6 compounds (amitryptiline, sulfamethazine, sulfamethizole, sulfachloropyridazine, sulfadimethoxine, coumarin-314) was run as quality control every 8 samples to ensure consistent instrument and column performance.

MS^3^ experiments for further structural analysis of the metabolite feature with *m/z* 339.083 annotated as methylated pyochelin (**1**) were performed using a Waters Corporation Cortecs UPLC T3 column (2.1 × 150 mm, 1.6 µm particle size) coupled to a high-resolution accurate mass Orbitrap ID-X tribrid mass spectrometer. The chromatographic method for sample analysis involved elution with 100% water 0.1% formic acid (mobile phase A) and MeCN and 0.1% formic acid (mobile phase B) using the following gradient program: 0 min 95% A; 0.5 min 95% A; 6 min 0% A (curve 7); 9.4 min 0% A; 9.5 min 95% A; 11 min 95% A. The flow rate was set at 0.4 mL/min. The column temperature was set to 40 °C, and the injection volume was 1 µL. The Orbitrap ID-X is a tribrid spectrometer that utilizes quadrupole isolation with dual detectors, an orbitrap and an ion trap, with a maximum resolving power of 500,000 full width at half maximum (FWHM) at *m/z* 200 and mass accuracy of < 1 ppm. The heated electrospray ionization (HESI) source was operated at a vaporizer temperature of 275 °C, a spray voltage of 3.5 kV, and sheath, auxiliary, and sweep gas flows of 40, 8, and 1 in arbitrary units, respectively. The instrument acquired full MS data in the 100–1000 *m/z* range in positive ionization mode at 30,000 resolution. MS^3^ data was collected with a MS^1^ isolation window of 0.8 mz, HCD activation of 30% ± 50%, a MS^2^ isolation window of 1.8 mz, MS^2^ HCD activation of 40%, and product ion detection in the orbitrap at 30,000 resolution.

### Data processing, feature-based molecular networking, and feature annotation

The LC–MS/MS data presented in this manuscript is deposited in the online repository MassIVE and publicly available (MSV000087793). The Compass DataAnalysis software was used to convert all the raw spectral files (.d format) to centroided, lock-mass corrected format (.mzXML) for downstream analyses. The converted spectral files were uploaded to MZmine2 (v.2.5.1) for feature detection^44^. Data files were batch processed and filtered using a MS^1^ mass detection signal threshold of 5.0 × 10^2^ counts. The following parameters were applied: (i) chromatogram builder (minimum time span: 0.05 min; minimum intensity of the highest data point in the chromatogram: 1.5 × 10^3^; *m/z* tolerance: 10 ppm); (ii) chromatogram deconvolution (local minimum search, *m/z* range for MS^2^ scan pairing: 0.025 Da; retention time range for MS^2^ scan pairing: 0.2 min); (iii) isotopic peaks grouper (*m/z* tolerance: 10 ppm; retention time tolerance absolute: 0.1 min; maximum charge: + 3; monotonic shape: true; representative isotope: most intense); (iv) join aligner (*m/z* tolerance: 10 ppm; retention time tolerance: 0.1 min); (v) peak finder (intensity tolerance: 10%; retention time tolerance (absolute): 0.1; *m/z* tolerance: 10 ppm). A table with ion intensities for each feature was exported (.csv format) for statistical analyses and the “Export for SIRIUS” module was used to generate an .mgf file for batch analysis with SIRUS 4. Additionally, the “Export for GNPS” module was used to convert and export the feature quantification table (.csv format) and the corresponding list of MS^2^ spectra linked to MS^1^ features (.mgf format) needed to generate a feature-based molecular network. The feature quantitation table and .mgf file were submitted to the Global Natural Products Molecular Networking (GNPS) platform along with a metadata file (.txt format) containing sample information, and a feature-based molecular network was created^45^. The molecular network and parameters used can be accessed using the following link: https://gnps.ucsd.edu/ProteoSAFe/status.jsp?task=a80b05b26a1747cfa6caf7f9446edd75. Briefly, the data was filtered by removing all MS/MS peaks within ± 17 Da of the precursor *m/z*. A parent mass tolerance of 0.01 Da and a MS/MS fragment ion tolerance of 0.05 Da were applied to create consensus spectra. The network was created with the edges filtered to have a cosine score above 0.7 and at least 4 matched peaks, and edges connecting two nodes were set to be kept in the network if each of the nodes appeared in each other's respective top 10 most similar nodes. All the mass spectra in the generated network were queried against the spectral libraries available on GNPS. The matched experimental and library spectra were set to have a similarity score above 0.7 and more than 4 matched peaks. This network was exported to Cytoscape for visualization, and nodes appearing in media or solvent blanks were removed for clarity (unless otherwise indicated)^120^. Boxplots were constructed for features of interest using the Plotter Dashboard (v.0.4) available on the GNPS platform. The UpSet plot was generated with the UpSetR package in R^48^.

Metabolomic features of interest that remained unknown after library searching were further annotated by first searching through literature for molecules which are known to be produced by *Burkholderia* spp. and developing an in-house database. Our database was curated by inserting structures manipulated using MarvinSketch (v.20.9.0, ChemAxon Ltd.) into a spreadsheet installed with JChem for Excel (v.20.8.0.62, ChemAxon Ltd.). This database was used to identify candidate annotations for features, and these annotations were confirmed by manually inspecting MS^2^ spectra and either comparing against published MS^2^ spectra (when available) or against the MS^2^ spectra of commercial analytical standards. We then applied the MS2LDA workflow to recognize patterns of common MS^2^ fragments and neutral losses (“Mass2Motifs”) corresponding to molecular substructures in our dataset^121^. This MS2LDA analysis is available at the following link: https://gnps.ucsd.edu/ProteoSAFe/status.jsp?task=628622833d0046048dba64e65ced542c. In addition, we performed batch analysis on the dataset using SIRIUS 4 integrated with CSI:FingerID and CANOPUS^91,92,122–125^. SIRIUS 4 (v.4.0.1) was employed (using the default settings for a qTOF instrument) to predict molecular formulas for unknown features and develop fragmentation trees for manual annotation of MS^2^ spectra^122–124^. CSI:FingerID was then utilized to predict molecular properties of unknown features, which were then queried against molecular properties predicted for compounds in all available molecular databases^125^. This in silico tool led to a ranked list of predicted structures, even when published MS^2^ spectra were not available for these structures. Next, we deployed CANOPUS to classify features into molecular families using ClassyFire, providing biological insight in the absence of structural annotations^91,92^. MASST searching was used to find metabolomic datasets collected by other researchers containing a match to the metabolic feature annotated as enantiothiazostatin (**1**)^67^. This MASST job and the parameters used are available at: https://gnps.ucsd.edu/ProteoSAFe/result.jsp?task=c5d7b5cc7459443286fed543e5140ca4&view=view_all_datasets_matched.

To determine differences in trimethoprim metabolism between *B. cenocepacia* strains grown in SCFM2 and LB media, metabolic features containing a trimethoprim substructure (appearing as Mass2Motif 543 in the MS2LDA analysis) were extracted from the feature quantification table (.csv format) generated by MZmine2. These features were then subjected to further statistical analysis on the MetaboAnalyst web server^126^. After uploading the peak intensity table for trimethoprim metabolites, data was log transformed and Pareto scaling was applied. The volcano plot comparing trimethoprim against non-trimethoprim cultures were then created using a fold-change threshold of 2.0 and an FDR adjusted p value threshold of 0.05, as calculated by a t-test.

### ICP-MS analysis of LB and SCFM2 media

ICP-MS was performed on aliquots of LB and SCFM2 media on a Perkin Elmer NexION 2000 with a S10 Autosampler at the Emory University Mass Spectrometry Center. Samples were diluted 1:10 and 1:100 with Type I deionized water before aspirating into the plasma using the NexION 2000 Peristaltic pump. A kinetic energy discrimination method with helium flow set to 5 arbitrary units was used to detect the ^57^Fe isotope using a pulse counting detection method. Calibration was done from 0.1 to 1000 ppb. Samples were analyzed in technical triplicate for each media type. To determine the amount of iron in each media sample, the mean and standard deviation of all concentrations measured across both dilutions was calculated and converted from ppb to micromolar units.

## Supplementary Information

Supplementary Information.

## Data Availability

The data obtained in this study has been deposited with the Mass Spectrometry Interactive Virtual Environment (MassIVE) with the identifier MSV000087793 and is accessible at ftp://massive.ucsd.edu/MSV000087793/.

## References

[1] Lyczak JB, Cannon CL, Pier GB (2002). Lung infections associated with cystic fibrosis. Clin. Microbiol. Rev..

[2] Turcios NL (2020). Cystic fibrosis lung disease: An overview. Respir. Care.

[3] Lipuma JJ (2005). Update on the *Burkholderia cepacia* complex. Curr. Opin. Pulm. Med..

[4] Lipuma JJ (2003). *Burkholderia cepacia* complex as human pathogens. J. Nematol..

[5] Rhodes KA, Schweizer HP (2016). Antibiotic resistance in *Burkholderia* species. Drug Resist. Updat..

[6] Scoffone VC (2017). *Burkholderia cenocepacia* infections in cystic fibrosis patients: Drug resistance and therapeutic approaches. Front. Microbiol..

[7] LiPuma JJ, Dasen SE, Nielson DW, Stern RC, Stull TL (1990). Person-to-person transmission of *Pseudomonas cepacia* between patients with cystic fibrosis. Lancet.

[8] Govan JR, Brown AR, Jones AM (2007). Evolving epidemiology of *Pseudomonas aeruginosa* and the *Burkholderia cepacia* complex in cystic fibrosis lung infection. Future Microbiol..

[9] Mahenthiralingam E, Urban TA, Goldberg JB (2005). The multifarious, multireplicon *Burkholderia cepacia* complex. Nat. Rev. Microbiol..

[10] LiPuma JJ (2001). Disproportionate distribution of *Burkholderia cepacia* complex species and transmissibility markers in cystic fibrosis. Am. J. Respir. Crit. Care Med..

[11] Drevinek P, Mahenthiralingam E (2010). *Burkholderia cenocepacia* in cystic fibrosis: Epidemiology and molecular mechanisms of virulence. Clin. Microbiol. Infect..

[12] Courtney JM (2004). Clinical outcome of *Burkholderia cepacia* complex infection in cystic fibrosis adults. J. Cyst. Fibros..

[13] Jones AM (2004). *Burkholderia cenocepacia* and *Burkholderia multivorans*: Influence on survival in cystic fibrosis. Thorax.

[14] Zlosnik JE (2015). *Burkholderia* species infections in patients with cystic fibrosis in British Columbia, Canada. 30 years' experience. Ann. Am. Thorac. Soc..

[15] De Soyza A (2010). Lung transplantation for patients with cystic fibrosis and *Burkholderia cepacia* complex infection: A single-center experience. J. Heart Lung Transplant..

[16] Liu X, Cheng YQ (2014). Genome-guided discovery of diverse natural products from *Burkholderia* sp. J. Ind. Microbiol. Biotechnol..

[17] Uehlinger S (2009). Identification of specific and universal virulence factors in *Burkholderia cenocepacia* strains by using multiple infection hosts. Infect. Immun..

[18] Kunakom S, Eustaquio AS (2019). *Burkholderia* as a source of natural products. J. Nat. Prod..

[19] Bodilis J (2018). Comparative genomics of environmental and clinical *Burkholderia cenocepacia* strains closely related to the highly transmissible epidemic ET12 Lineage. Front. Microbiol..

[20] Sousa SA, Feliciano JR, Pita T, Guerreiro SI, Leitao JH (2017). *Burkholderia cepacia* complex regulation of virulence gene expression: A review. Genes.

[21] Schroter L, Dersch P (2019). Phenotypic diversification of microbial pathogens-cooperating and preparing for the future. J. Mol. Biol..

[22] Neve RL, Carrillo BD, Phelan VV (2021). Commercial porcine gastric mucin contributes to variation in production of small molecule virulence factors by *Pseudomonas aeruginosa* when cultured in different formulations of artificial sputum medium. bioRxiv.

[23] Palmer KL, Aye LM, Whiteley M (2007). Nutritional cues control *Pseudomonas aeruginosa* multicellular behavior in cystic fibrosis sputum. J. Bacteriol..

[24] Palmer KL, Mashburn LM, Singh PK, Whiteley M (2005). Cystic fibrosis sputum supports growth and cues key aspects of *Pseudomonas aeruginosa* physiology. J. Bacteriol..

[25] Drevinek P (2008). Gene expression changes linked to antimicrobial resistance, oxidative stress, iron depletion and retained motility are observed when *Burkholderia cenocepacia* grows in cystic fibrosis sputum. BMC Infect. Dis..

[26] Fung C (2010). Gene expression of *Pseudomonas aeruginosa* in a mucin-containing synthetic growth medium mimicking cystic fibrosis lung sputum. J. Med. Microbiol..

[27] Hare NJ (2012). Proteomics of *Pseudomonas aeruginosa* Australian epidemic strain 1 (AES-1) cultured under conditions mimicking the cystic fibrosis lung reveals increased iron acquisition via the siderophore pyochelin. J. Proteome Res..

[28] Kirchner S (2012). Use of artificial sputum medium to test antibiotic efficacy against *Pseudomonas aeruginosa* in conditions more relevant to the cystic fibrosis lung. J. Vis. Exp.

[29] Quinn RA (2015). A Winogradsky-based culture system shows an association between microbial fermentation and cystic fibrosis exacerbation. ISME J..

[30] Sriramulu DD, Lunsdorf H, Lam JS, Romling U (2005). Microcolony formation: A novel biofilm model of *Pseudomonas aeruginosa* for the cystic fibrosis lung. J. Med. Microbiol..

[31] Turner KH, Wessel AK, Palmer GC, Murray JL, Whiteley M (2015). Essential genome of *Pseudomonas aeruginosa* in cystic fibrosis sputum. Proc. Natl. Acad. Sci. USA.

[32] Darch SE (2018). Spatial determinants of quorum signaling in a *Pseudomonas aeruginosa* infection model. Proc. Natl. Acad. Sci. USA.

[33] Ghorbani P (2015). Short-chain fatty acids affect cystic fibrosis airway inflammation and bacterial growth. Eur. Respir. J..

[34] LaBauve AE, Wargo MJ (2014). Detection of host-derived sphingosine by *Pseudomonas aeruginosa* is important for survival in the murine lung. PLoS Pathog..

[35] Son MS, Matthews WJ, Kang Y, Nguyen DT, Hoang TT (2007). In vivo evidence of *Pseudomonas aeruginosa* nutrient acquisition and pathogenesis in the lungs of cystic fibrosis patients. Infect. Immun..

[36] Flynn JM, Niccum D, Dunitz JM, Hunter RC (2016). Evidence and role for bacterial mucin degradation in cystic fibrosis airway disease. PLoS Pathog..

[37] Schwab U (2014). Localization of *Burkholderia cepacia* complex bacteria in cystic fibrosis lungs and interactions with *Pseudomonas aeruginosa* in hypoxic mucus. Infect. Immun..

[38] Sanders NN, Van Rompaey E, De Smedt SC, Demeester J (2001). Structural alterations of gene complexes by cystic fibrosis sputum. Am. J. Respir. Crit. Care Med..

[39] Cornforth DM, Diggle FL, Melvin JA, Bomberger JM, Whiteley M (2020). Quantitative framework for model evaluation in microbiology research using *pseudomonas aeruginosa* and cystic fibrosis infection as a test case. MBio.

[40] Sfeir MM (2018). *Burkholderia cepacia* complex infections: More complex than the bacterium name suggest. J. Infect..

[41] Okada BK, Seyedsayamdost MR (2017). Antibiotic dialogues: Induction of silent biosynthetic gene clusters by exogenous small molecules. FEMS Microbiol. Rev..

[42] Okada BK, Wu Y, Mao D, Bushin LB, Seyedsayamdost MR (2016). Mapping the trimethoprim-induced secondary metabolome of *Burkholderia thailandensis*. ACS Chem. Biol..

[43] McAvoy AC (2020). Differences in cystic fibrosis-associated *Burkholderia* spp. bacteria metabolomes after exposure to the antibiotic trimethoprim. ACS Infect. Dis..

[44] Pluskal T, Castillo S, Villar-Briones A, Oresic M (2010). MZmine 2: Modular framework for processing, visualizing, and analyzing mass spectrometry-based molecular profile data. BMC Bioinform..

[45] Nothias LF (2020). Feature-based molecular networking in the GNPS analysis environment. Nat. Methods.

[46] Gonyar LA, Fankhauser SC, Goldberg JB (2015). Single amino acid substitution in homogentisate 1,2-dioxygenase is responsible for pigmentation in a subset of *Burkholderia cepacia* complex isolates. Environ. Microbiol. Rep..

[47] Keith KE, Killip L, He P, Moran GR, Valvano MA (2007). *Burkholderia cenocepacia* C5424 produces a pigment with antioxidant properties using a homogentisate intermediate. J. Bacteriol..

[48] Conway JR, Lex A, Gehlenborg N (2017). UpSetR: An R package for the visualization of intersecting sets and their properties. Bioinformatics.

[49] Lee AH (2017). Phenotypic diversity and genotypic flexibility of *Burkholderia cenocepacia* during long-term chronic infection of cystic fibrosis lungs. Genome Res..

[50] Malesevic M (2017). Virulence traits associated with *Burkholderia cenocepacia* ST856 epidemic strain isolated from cystic fibrosis patients. Antimicrob. Resist. Infect. Control.

[51] Moreira AS (2017). Variation of *Burkholderia cenocepacia* virulence potential during cystic fibrosis chronic lung infection. Virulence.

[52] Lieberman TD (2011). Parallel bacterial evolution within multiple patients identifies candidate pathogenicity genes. Nat. Genet..

[53] Skaar EP (2010). The battle for iron between bacterial pathogens and their vertebrate hosts. PLoS Pathog..

[54] Butt AT, Thomas MS (2017). Iron acquisition mechanisms and their role in the virulence of *Burkholderia* Species. Front. Cell Infect. Microbiol..

[55] Reid DW, Withers NJ, Francis L, Wilson JW, Kotsimbos TC (2002). Iron deficiency in cystic fibrosis: Relationship to lung disease severity and chronic *Pseudomonas aeruginosa* infection. Chest.

[56] Stites SW, Plautz MW, Bailey K, O'Brien-Ladner AR, Wesselius LJ (1999). Increased concentrations of iron and isoferritins in the lower respiratory tract of patients with stable cystic fibrosis. Am. J. Respir. Crit. Care Med..

[57] Ellermann M, Arthur JC (2017). Siderophore-mediated iron acquisition and modulation of host-bacterial interactions. Free Radic. Biol. Med..

[58] Meyer JM, Hohnadel D, Halle F (1989). Cepabactin from *Pseudomonas cepacia*, a new type of siderophore. J. Gen. Microbiol..

[59] Darling P, Chan M, Cox AD, Sokol PA (1998). Siderophore production by cystic fibrosis isolates of *Burkholderia cepacia*. Infect. Immun..

[60] Lybbert AC, Williams JL, Raghuvanshi R, Jones AD, Quinn RA (2020). Mining public mass spectrometry data to characterize the diversity and ubiquity of *P. aeruginosa* specialized metabolites. Metabolites.

[61] Cox CD, Rinehart KL, Moore ML, Cook JC (1981). Pyochelin: Novel structure of an iron-chelating growth promoter for *Pseudomonas aeruginosa*. Proc. Natl. Acad. Sci. USA.

[62] Shindo K, Takenaka A, Noguchi T, Hayakawa Y, Seto H (1989). Thiazostatin A and thiazostatin B, new antioxidants produced by *Streptomyces tolurosus*. J. Antibiot..

[63] Schlegel K, Taraz K, Budzikiewicz H (2004). The stereoisomers of pyochelin, a siderophore of *Pseudomonas aeruginosa*. Biometals.

[64] Inahashi Y (2017). Watasemycin biosynthesis in *Streptomyces venezuelae*: Thiazoline C-methylation by a type B radical-SAM methylase homologue. Chem. Sci..

[65] Ahmadi MK, Fawaz S, Jones CH, Zhang G, Pfeifer BA (2015). Total biosynthesis and diverse applications of the nonribosomal peptide-polyketide siderophore yersiniabactin. Appl. Environ. Microbiol..

[66] Miller DA, Walsh CT, Luo L (2001). C-methyltransferase and cyclization domain activity at the intraprotein PK/NRP switch point of yersiniabactin synthetase. J. Am. Chem. Soc..

[67] Wang M (2020). Mass spectrometry searches using MASST. Nat. Biotechnol..

[68] Xu G, Guo H, Lv H (2019). Metabolomics assay identified a novel virulence-associated siderophore encoded by the high-pathogenicity island in uropathogenic *Escherichia coli*. J. Proteome Res..

[69] Braud A, Hannauer M, Mislin GL, Schalk IJ (2009). The *Pseudomonas aeruginosa* pyochelin-iron uptake pathway and its metal specificity. J. Bacteriol..

[70] Yamada Y, Seki N, Kitahara T, Takahashi M, Matsui M (1970). The structure and synthesis of aeruginoic acid (2-o-hydroxy-phenylthiazole-4-carboxylic acid). Agric. Biol. Chem..

[71] Kaplan AR, Musaev DG, Wuest WM (2021). Pyochelin biosynthetic metabolites bind iron and promote growth in *Pseudomonads* demonstrating siderophore-like activity. ACS Infect. Dis..

[72] Lee J (2013). A cell-cell communication signal integrates quorum sensing and stress response. Nat. Chem. Biol..

[73] Cornelis P (2020). Putting an end to the *Pseudomonas aeruginosa* IQS controversy. Microbiologyopen.

[74] Trottmann F, Franke J, Ishida K, Garcia-Altares M, Hertweck C (2019). A pair of bacterial siderophores releases and traps an intercellular signal molecule: An unusual case of natural nitrone bioconjugation. Angew. Chem. Int. Ed. Engl..

[75] Lacerna NM (2019). Mindapyrroles A-C, pyoluteorin analogues from a shipworm-associated bacterium. J. Nat. Prod..

[76] Lee JY, Moon SS, Hwang BK (2003). Isolation and antifungal and antioomycete activities of aerugine produced by *Pseudomonas fluorescens* strain MM-B16. Appl. Environ. Microbiol..

[77] Malott RJ (2009). A *Burkholderia cenocepacia* orphan LuxR homolog is involved in quorum-sensing regulation. J. Bacteriol..

[78] Sokol PA (1986). Production and utilization of pyochelin by clinical isolates of *Pseudomonas cepacia*. J. Clin. Microbiol..

[79] Mukherjee S, Bassler BL (2019). Bacterial quorum sensing in complex and dynamically changing environments. Nat. Rev. Microbiol..

[80] Whiteley M, Diggle SP, Greenberg EP (2017). Progress in and promise of bacterial quorum sensing research. Nature.

[81] Huber B (2001). The *cep* quorum-sensing system of *Burkholderia cepacia* H111 controls biofilm formation and swarming motility. Microbiology.

[82] Chandler JR (2009). Mutational analysis of *Burkholderia thailandensis* quorum sensing and self-aggregation. J. Bacteriol..

[83] Scoffone VC, Trespidi G, Chiarelli LR, Barbieri G, Buroni S (2019). Quorum sensing as antivirulence target in cystic fibrosis pathogens. Int. J. Mol. Sci..

[84] Suppiger A, Schmid N, Aguilar C, Pessi G, Eberl L (2013). Two quorum sensing systems control biofilm formation and virulence in members of the *Burkholderia cepacia* complex. Virulence.

[85] Subramoni S, Sokol PA (2012). Quorum sensing systems influence *Burkholderia cenocepacia* virulence. Future Microbiol..

[86] Lewenza S, Conway B, Greenberg EP, Sokol PA (1999). Quorum sensing in *Burkholderia cepacia*: Identification of the LuxRI homologs CepRI. J. Bacteriol..

[87] Malott RJ, Baldwin A, Mahenthiralingam E, Sokol PA (2005). Characterization of the *cciIR* quorum-sensing system in *Burkholderia cenocepacia*. Infect. Immun..

[88] Ryan GT, Wei Y, Winans SC (2013). A LuxR-type repressor of *Burkholderia cenocepacia* inhibits transcription via antiactivation and is inactivated by its cognate acylhomoserine lactone. Mol. Microbiol..

[89] Patel NM, Moore JD, Blackwell HE, Amador-Noguez D (2016). Identification of unanticipated and novel *N*-acyl l-homoserine lactones (AHLs) using a sensitive non-targeted LC-MS/MS method. PLoS ONE.

[90] Jenul C (2018). Biosynthesis of fragin is controlled by a novel quorum sensing signal. Nat. Commun..

[91] Dührkop K (2020). Systematic classification of unknown metabolites using high-resolution fragmentation mass spectra. Nat. Biotechnol..

[92] Djoumbou Feunang Y (2016). ClassyFire: Automated chemical classification with a comprehensive, computable taxonomy. J. Cheminform..

[93] Kretsch AM (2018). Discovery of (dihydro)pyrazine N-oxides via genome mining in *Pseudomonas*. Org. Lett..

[94] Vallet-Gely I, Opota O, Boniface A, Novikov A, Lemaitre B (2010). A secondary metabolite acting as a signalling molecule controls *Pseudomonas entomophila* virulence. Cell Microbiol..

[95] Opota O (2011). Monalysin, a novel ß-pore-forming toxin from the *Drosophila* pathogen *Pseudomonas entomophila*, contributes to host intestinal damage and lethality. PLoS Pathog..

[96] Carrion VJ (2014). Mangotoxin production of *Pseudomonas syringae* pv. syringae is regulated by MgoA. BMC Microbiol..

[97] Weisskopf L, Schulz S, Garbeva P (2021). Microbial volatile organic compounds in intra-kingdom and inter-kingdom interactions. Nat. Rev. Microbiol..

[98] Sohlenkamp C, Geiger O (2016). Bacterial membrane lipids: Diversity in structures and pathways. FEMS Microbiol. Rev..

[99] Talbot HM (2016). Analysis of non-derivatised bacteriohopanepolyols by ultrahigh-performance liquid chromatography/tandem mass spectrometry. Rapid Commun. Mass Spectrom..

[100] Talbot HM, Rohmer M, Farrimond P (2007). Structural characterisation of unsaturated bacterial hopanoids by atmospheric pressure chemical ionisation liquid chromatography/ion trap mass spectrometry. Rapid Commun. Mass Spectrom..

[101] Schmerk CL, Bernards MA, Valvano MA (2011). Hopanoid production is required for low-pH tolerance, antimicrobial resistance, and motility in *Burkholderia cenocepacia*. J. Bacteriol..

[102] Schmerk CL (2015). Elucidation of the *Burkholderia cenocepacia* hopanoid biosynthesis pathway uncovers functions for conserved proteins in hopanoid-producing bacteria. Environ. Microbiol..

[103] Cvejic JH (2000). Bacterial triterpenoids of the hopane series as biomarkers for the chemotaxonomy of *Burkholderia*, *Pseudomonas* and *Ralstonia* spp.. FEMS Microbiol. Lett..

[104] Simonin P, Tindall B, Rohmer M (1994). Structure elucidation and biosynthesis of 31-methylhopanoids from *Acetobacter europaeus*. Studies on a new series of bacterial triterpenoids. Eur. J. Biochem..

[105] Vilcheze C, Llopiz P, Neunlist S, Poralla K, Rohmer M (1994). Prokaryotic triterpenoids: New hopanoids from the nitrogen-fixing bacteria *Azotobacter vinelandii,**Beijerinckia indica* and *Beijerinckia mobilis*. Microbiology.

[106] Zhao N (1996). Structures of two bacteriohopanoids with acyclic pentol side-chains from the cyanobacterium *Nostoc* PCC 6720. Tetrahedron.

[107] Ruskoski SA, Bullard JW, Champlin FR (2014). Cell envelope phospholipid composition of *Burkholderia multivorans*. Curr. Microbiol..

[108] Poger D, Mark AE (2015). A ring to rule them all: The effect of cyclopropane fatty acids on the fluidity of lipid bilayers. J. Phys. Chem. B.

[109] Palacios-Chaves L (2012). Identification and functional analysis of the cyclopropane fatty acid synthase of *Brucella abortus*. Microbiology.

[110] Vazquez L, Jordan A, Reglero G, Torres CF (2015). A first attempt into the production of acylglycerol mixtures from *Echium* oil. Front. Bioeng. Biotechnol..

[111] Bornscheuer U (1994). Lipase of *Pseudomonas cepacia* for biotechnological purposes: Purification, crystallization and characterization. Biochim. Biophys. Acta.

[112] O'Grady EP, Viteri DF, Malott RJ, Sokol PA (2009). Reciprocal regulation by the CepIR and CciIR quorum sensing systems in *Burkholderia cenocepacia*. BMC Genomics.

[113] Jumina J (2019). Preparation of monoacylglycerol derivatives from indonesian edible oil and their antimicrobial assay against *Staphylococcus aureus* and *Escherichia coli*. Sci. Rep..

[114] Yoon BK, Jackman JA, Valle-Gonzalez ER, Cho NJ (2018). Antibacterial Free fatty acids and monoglycerides: Biological activities, experimental testing, and therapeutic applications. Int. J. Mol. Sci..

[115] Cho G, Lee E, Kim J (2021). Structural insights into phosphatidylethanolamine formation in bacterial membrane biogenesis. Sci. Rep..

[116] Pi J, Wu X, Feng Y (2016). Fragmentation patterns of five types of phospholipids by ultra-high-performance liquid chromatography electrospray ionization quadrupole time-of-flight tandem mass spectrometry. Anal. Methods.

[117] Taylor CJ, Anderson AJ, Wilkinson SG (1998). Phenotypic variation of lipid composition in *Burkholderia cepacia*: A response to increased growth temperature is a greater content of 2-hydroxy acids in phosphatidylethanolamine and ornithine amide lipid. Microbiology.

[118] Govan JR (1993). Evidence for transmission of *Pseudomonas cepacia* by social contact in cystic fibrosis. Lancet.

[119] Mahenthiralingam E, Campbell ME, Henry DA, Speert DP (1996). Epidemiology of *Burkholderia cepacia* infection in patients with cystic fibrosis: Analysis by randomly amplified polymorphic DNA fingerprinting. J. Clin. Microbiol..

[120] Shannon P (2003). Cytoscape: A software environment for integrated models of biomolecular interaction networks. Genome Res..

[121] van der Hooft JJ, Wandy J, Barrett MP, Burgess KE, Rogers S (2016). Topic modeling for untargeted substructure exploration in metabolomics. Proc. Natl. Acad. Sci. USA.

[122] Bocker S, Duhrkop K (2016). Fragmentation trees reloaded. J. Cheminform..

[123] Bocker S, Letzel MC, Liptak Z, Pervukhin A (2009). SIRIUS: Decomposing isotope patterns for metabolite identification. Bioinformatics.

[124] Dührkop K (2019). SIRIUS 4: A rapid tool for turning tandem mass spectra into metabolite structure information. Nat. Methods.

[125] Dührkop K, Shen H, Meusel M, Rousu J, Böcker S (2015). Searching molecular structure databases with tandem mass spectra using CSI:FingerID. Proc. Natl. Acad. Sci. USA.

[126] Chong J, Wishart DS, Xia J (2019). Using MetaboAnalyst 4.0 for comprehensive and integrative metabolomics data analysis. Curr. Protocols Bioinform..

