# Supplementary material for: Metabolomic profiling of Burkholderia cenocepacia in synthetic cystic fibrosis sputum medium reveals nutrient environment-specific production of virulence factors
Source: Sci Rep. 2021 Nov 1;11:21419. doi: 10.1038/s41598-021-00421-4 (PMC8560942; doi:10.1038/s41598-021-00421-4)
Supplement: Supplementary file 1 — Supplementary Information. [file 41598_2021_421_MOESM1_ESM.pdf]

Supplementary Information

Metabolomic Profiling of *Burkholderia cenocepacia* strains in Synthetic Cystic Fibrosis Sputum Media Reveals Nutrient Environment-Specific Production of Virulence Factors

Olakunle A. Jaiyesimi [1,#], Andrew C. McAvoy [1,#], David N. Fogg [1], and Neha Garg [1,2,*]

[1] School of Chemistry and Biochemistry, Georgia Institute of Technology, 950 Atlantic Drive, Atlanta, GA, 30332-2000, USA

[2] Center for Microbial Dynamics and Infection, Georgia Institute of Technology, 311 Ferst Drive, ES&T, Atlanta, Georgia 30332, USA; neha.garg@chemistry.gatech.edu

* Correspondence: neha.garg@chemistry.gatech.edu

[#] Equal contribution

Supplementary information

Pages: 15

Figures: 12

Tables: 2

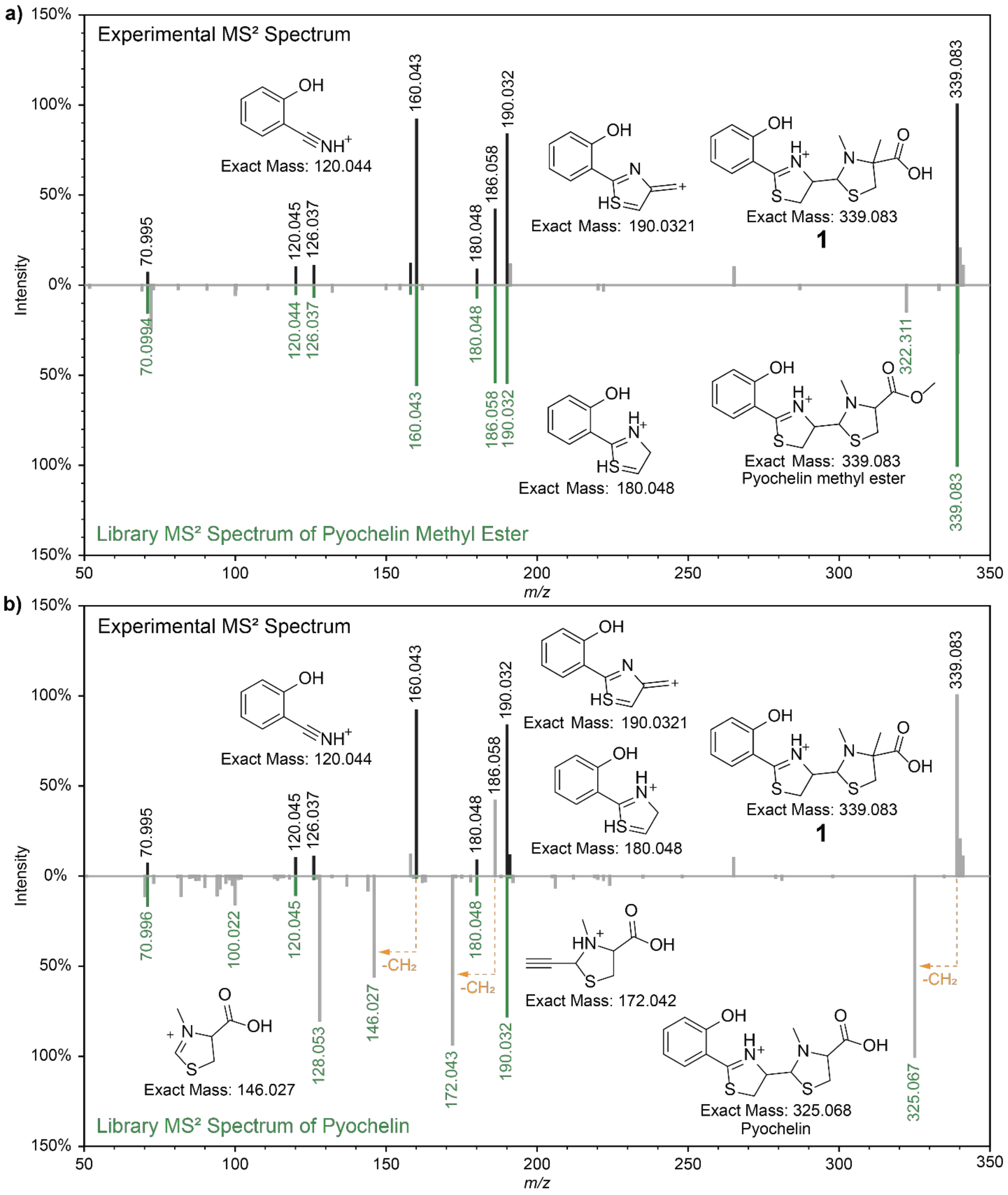

Supplementary Figure S1. a) Mirror plot comparing the experimental MS[2] spectrum (black trace) of methylated pyochelin (**1**) with its GNPS library hit to pyochelin methyl ester (green trace). **b)** Mirror plot comparing the experimental MS[2] spectrum (black trace) of the methylated pyochelin (**1**) with the GNPS library spectrum (green trace) of pyochelin, supporting structural similarity with the gain of a methyl group.

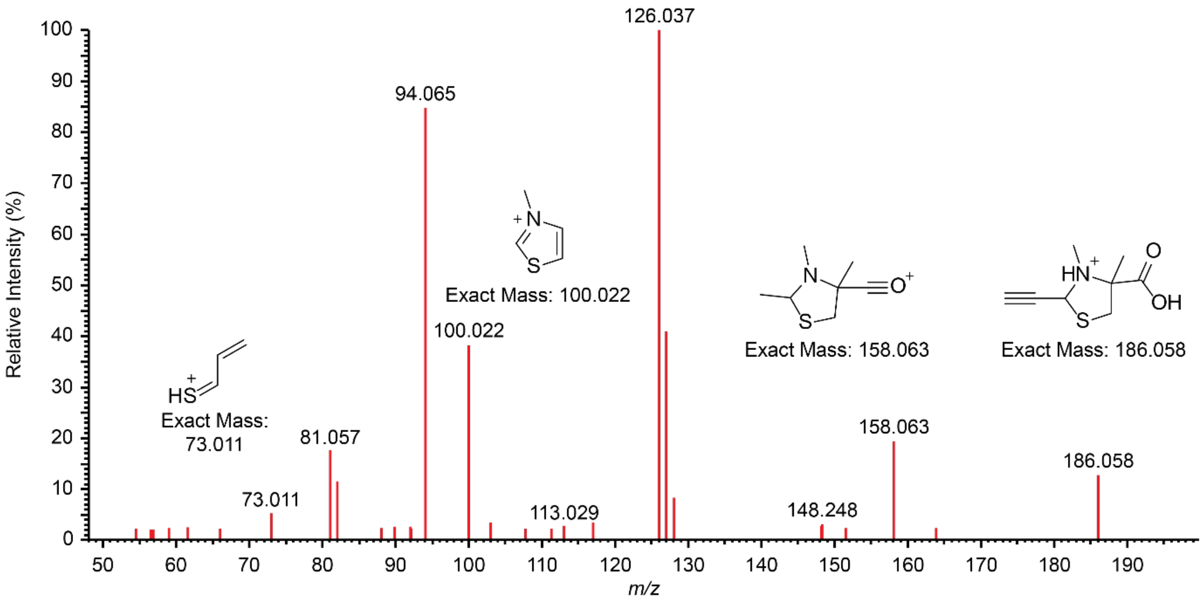

Supplementary Figure S2. Annotated MS^3 spectrum of the fragment ion with *m/z* 186.058 found in the MS^2 spectrum of the feature annotated as methylated pyochelin (**1**). The fragment at *m/z* 158.063 supports annotation of methylation at the C4" position of the thiazolidine ring.

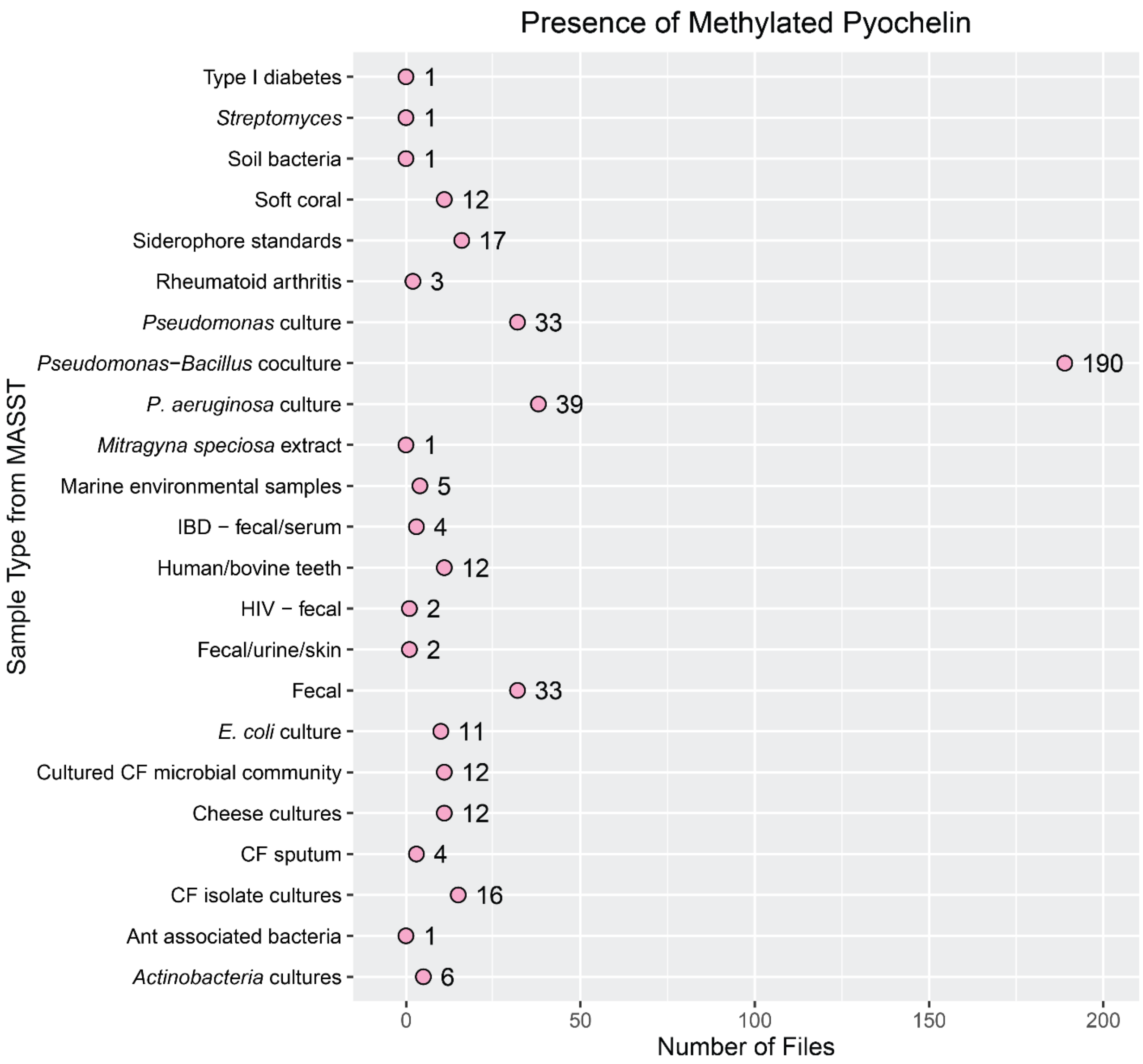

Supplementary Figure S3. Presence of methylated pyochelin (**1**) in publicly available metabolomics datasets as detected by MASST searching. The x-axis represents the number of files for each sample type containing at least one MS^2 spectrum with a match to the MS^2 of methylated pyochelin, while the y-axis indicates the sample type.

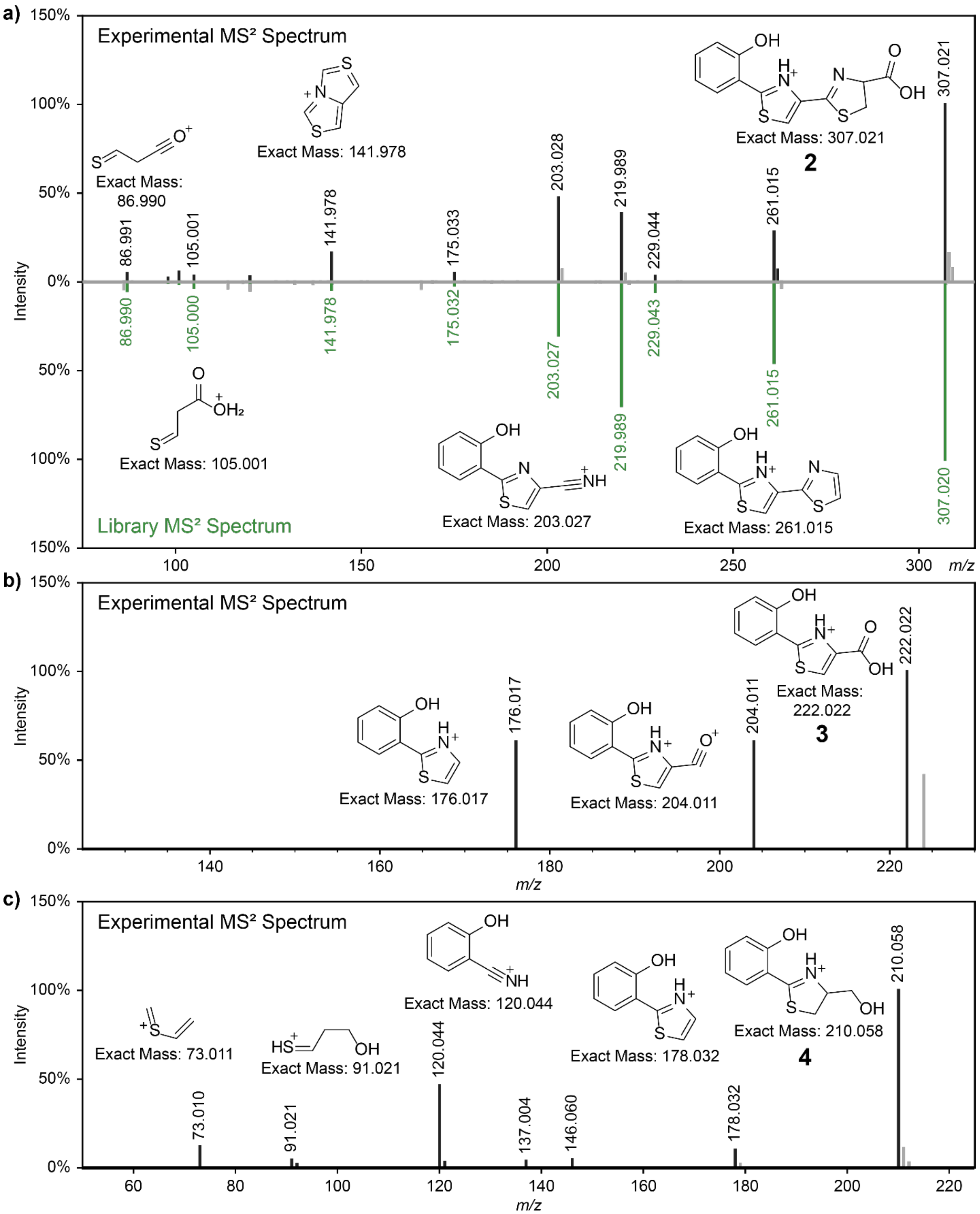

Supplementary Figure S4. a) Mirror plot of experimental MS^2 spectrum (black trace) and library spectrum (green trace) for feature annotated as HPTzTn-COOH (**2**). **b)** Annotated MS^2 spectrum for aeruginoic acid (**3**). **c)** Annotated MS^2 spectrum for aerugine (**4**).

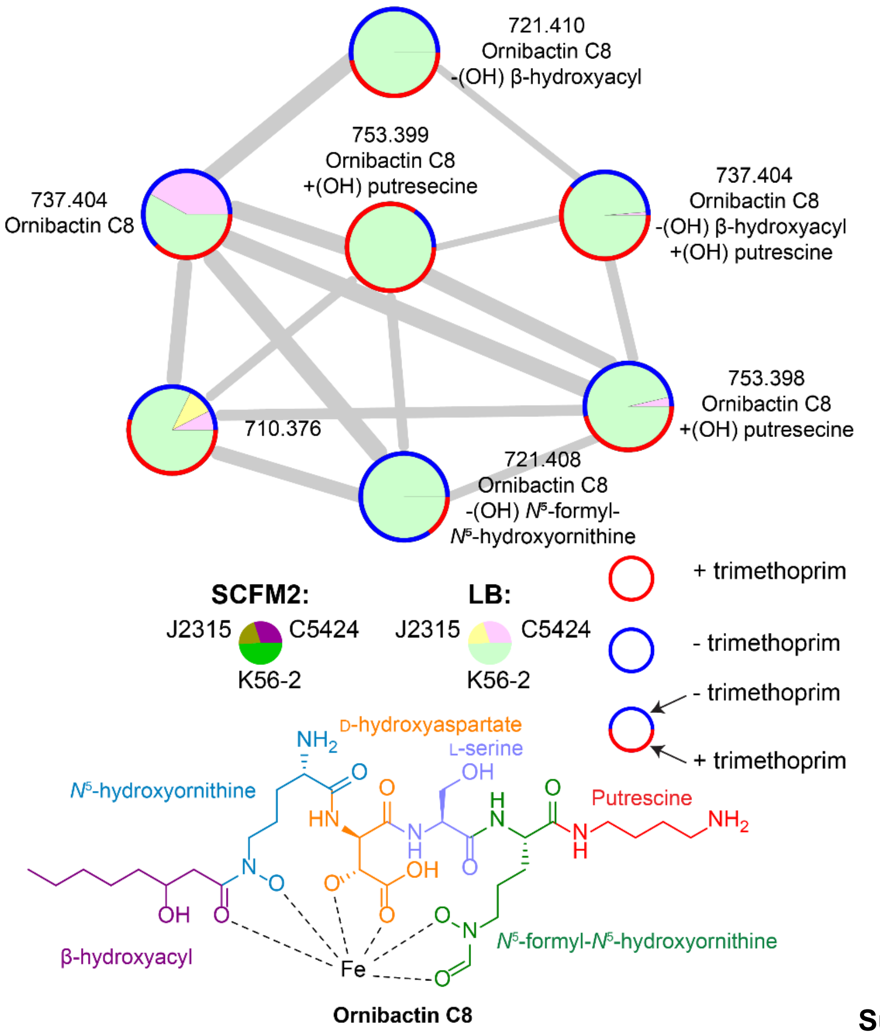

Supplementary Figure S5. Distribution of ornibactin C8 and related molecules across three *B. cenocepacia* strains. Pie charts within the nodes correspond to relative abundance in each strain (green: K56-2, purple: C5424, brown: J2315) and media condition (darker shade for SCFM2 and lighter shade for LB). The coloring of node border represents whether the metabolite was detected only in the presence of trimethoprim (red border), only in the absence of trimethoprim (blue), and both in the presence or absence of trimethoprim (mixed color). The border color (red vs. blue) corresponds to relative abundance in presence vs. absence of trimethoprim. Ornibactin C8 and analogs were only detected in cultures grown in LB, and not in SCFM2 media.

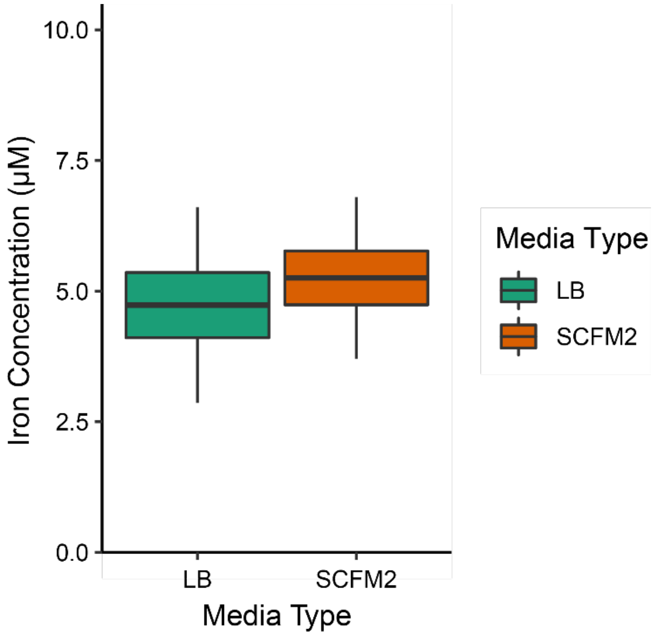

Supplementary Figure S6. Box and whisker plot of mean iron concentration in LB and SCFM2 media, as measured by ICP-MS. Outer edges of boxes represent one standard deviation from mean, outer edges of whiskers represent three standard deviations.

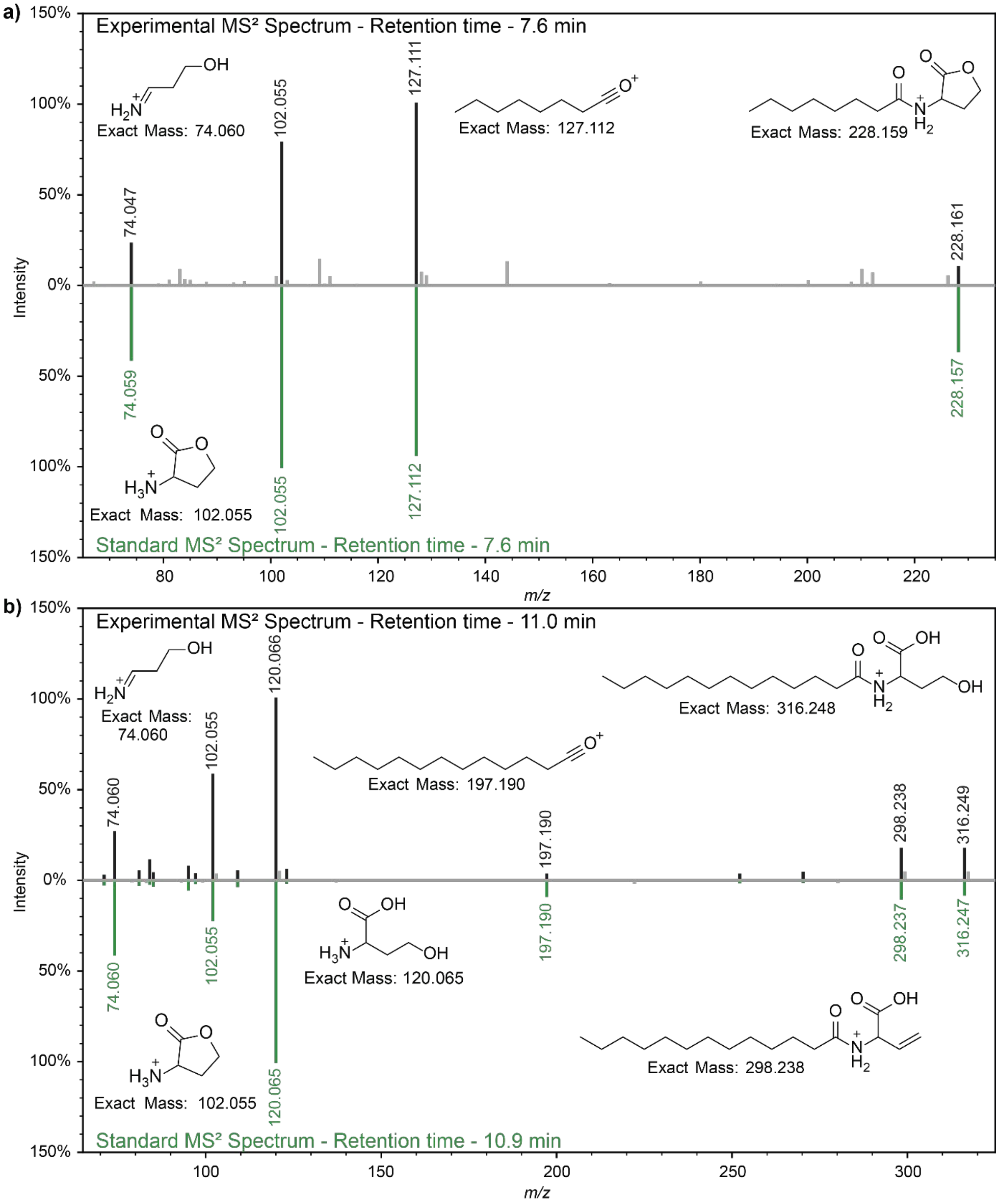

Supplementary Figure S7. a) Mirror plot of experimental MS^2 spectrum (black trace) and library spectrum (green trace) for feature annotated as C8-AHL (*m/z* 228.160, **5**) and **b)** hydrolyzed C13-AHL (*m/z* 316.249, **7**).

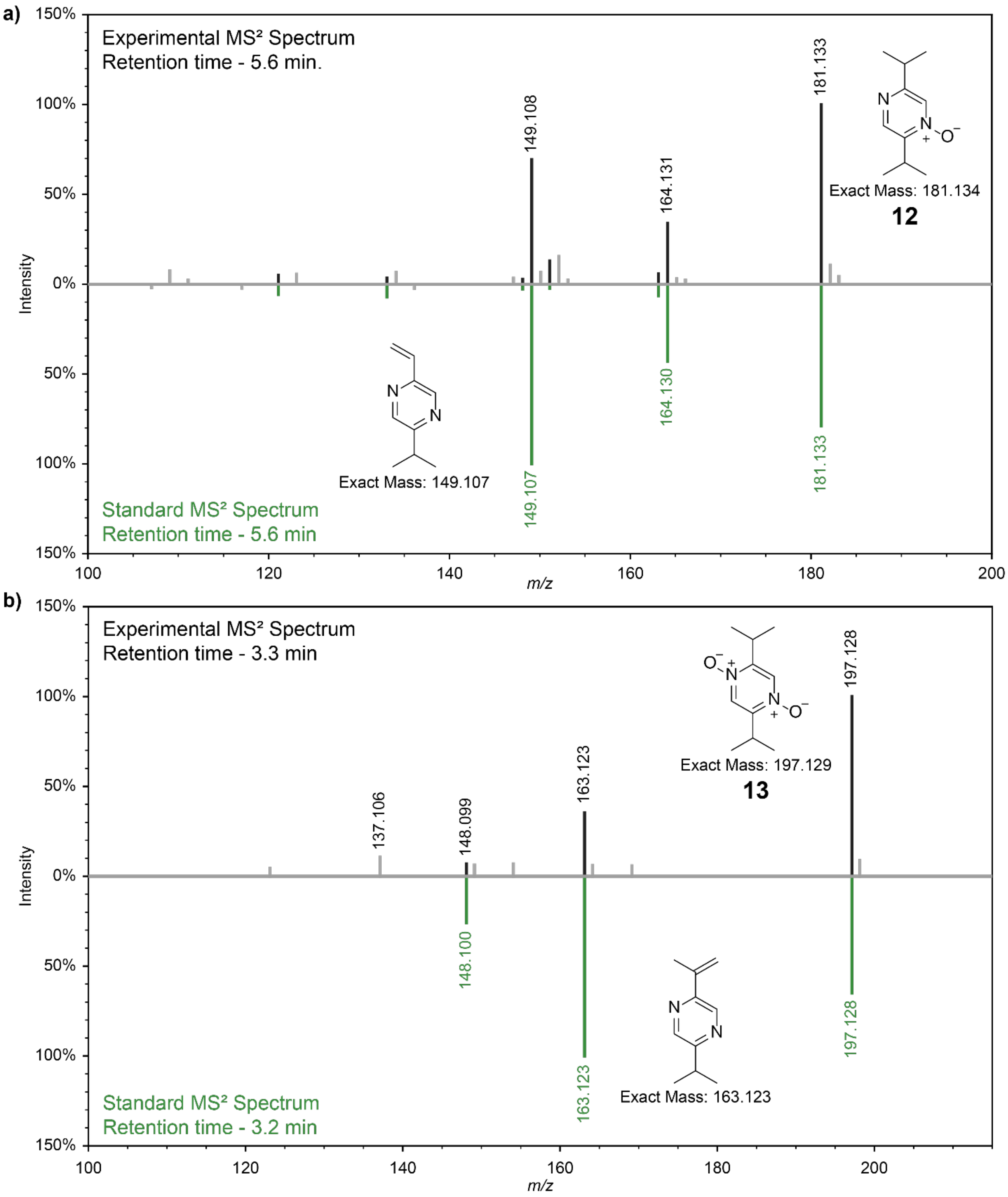

Supplementary Figure S8. Mirror plot MS^2 spectra of features annotated as **a)** PNO B (**12**) and **b)** PNO A (**13**) acquired on extracts of bacterial culture (black trace) and the MS^2 spectrum acquired from analysis of synthetic standards contributed by Kretsch *et. al* (green trace).

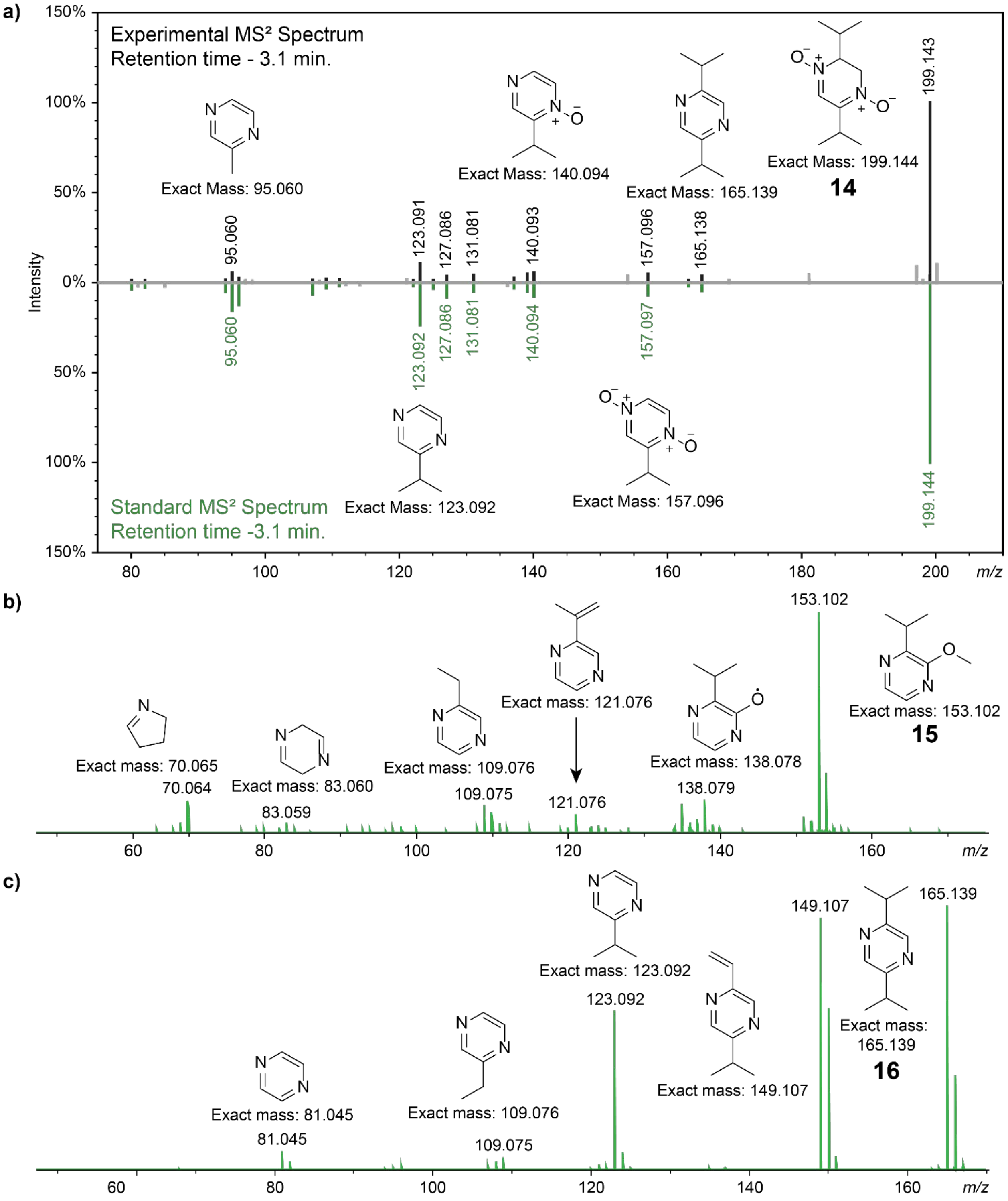

Supplementary Figure S9. The experimental MS^2 spectrum of features annotated as **a)** dPNO (**14**), **b)** 2-isopropyl-3-methoxypyrazine (**15**), and **c)** 2,5-diisopropylpyrazine (**16**) acquired on bacterial extracts. The MS^2 spectrum of dPNO was compared against data acquired on a synthetic standard contributed by Kretsch *et. al*, and is shown as a mirror plot in panel A.

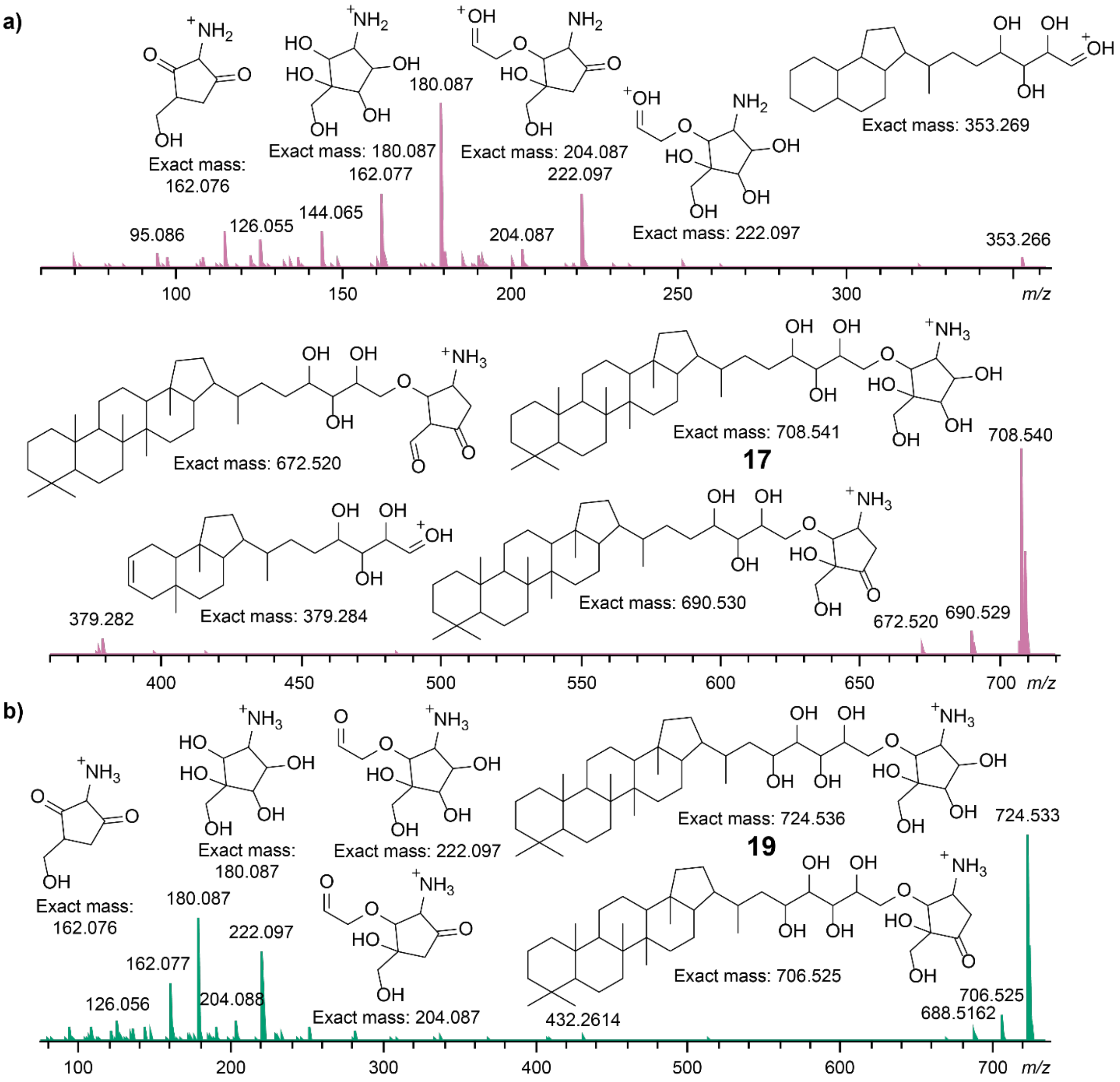

Supplementary Figure S10. The experimental MS[2] spectrum from features annotated as **a)** bacteriohopanetetrol (BHT, *m/z* 708.540) cyclitol ether (**17**) and **b)** bacteriohopanepentol (BHP, *m/z* 724.535) cyclitol ether (**19**) is shown.

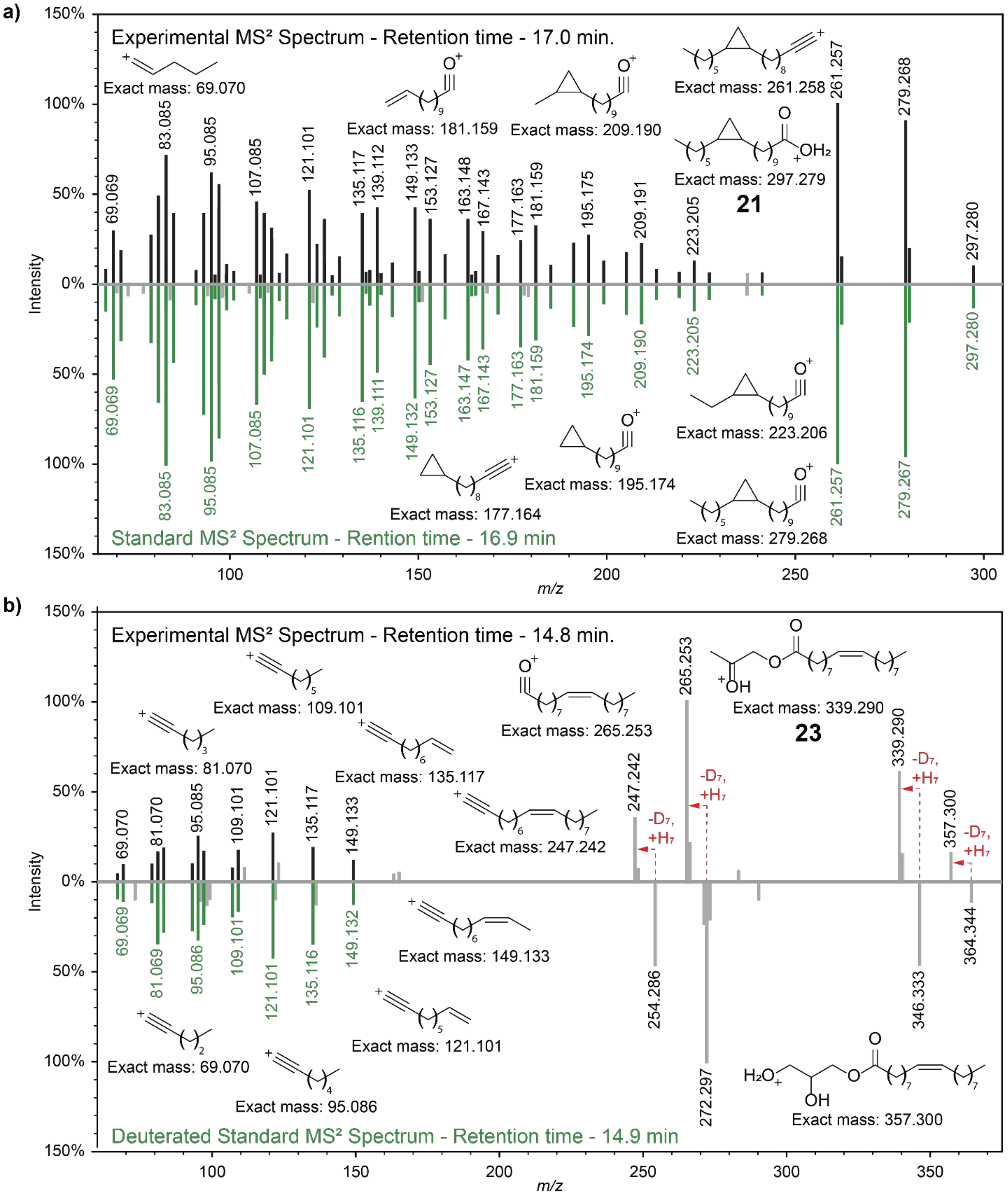

Supplementary Figure S11. The mirror plot of experimental MS^2 spectrum (black traces) of features annotated as **a)** phytomonic acid (**21**) and **b)** 18:1 MG (**23**) compared to experimental MS^2 spectrum (green traces) acquired from analysis of commercially acquired analytical standards.

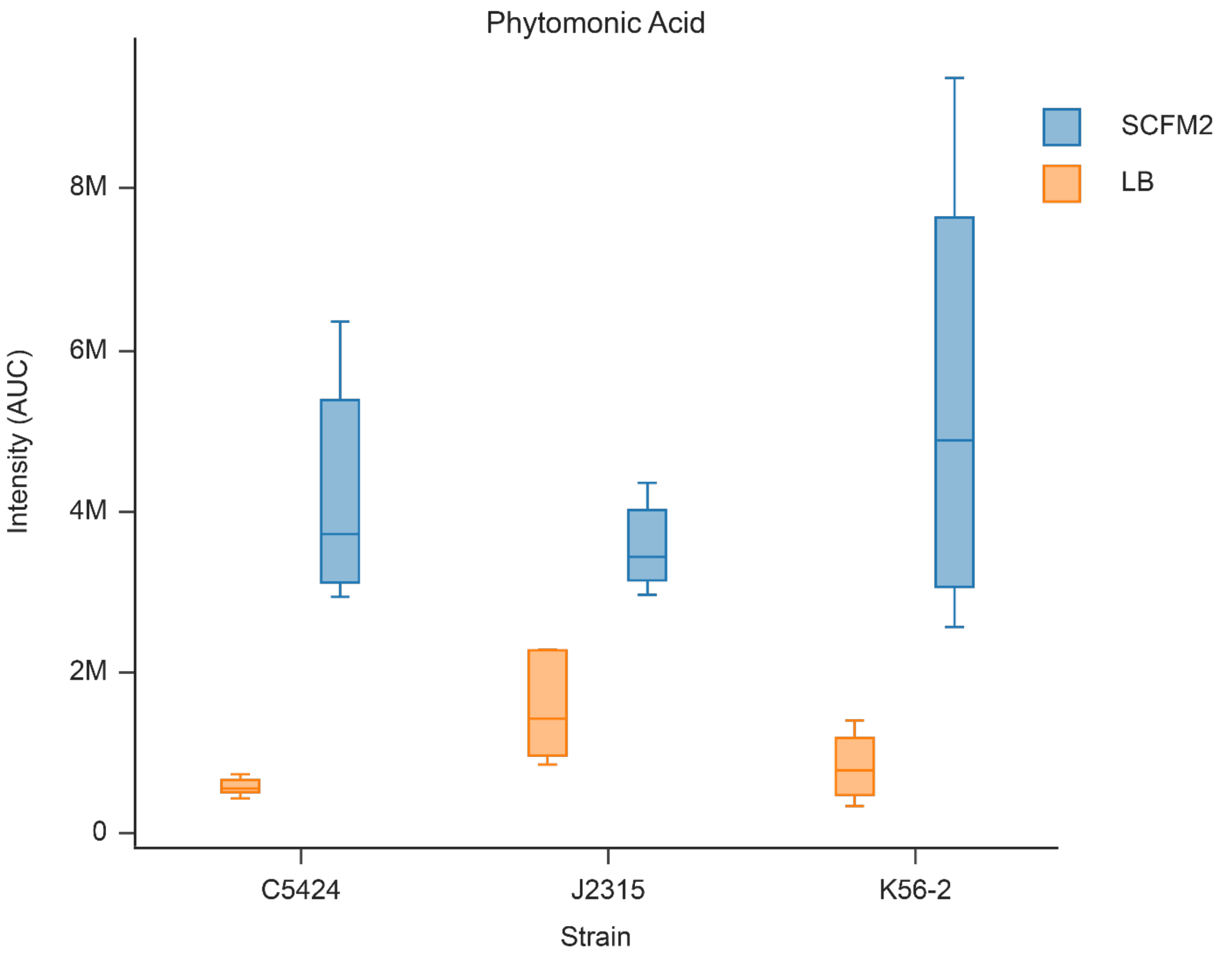

Supplementary Figure S12. Box-and-whisker plot showing relative abundance of phytomonic acid (**21**) across all three *B. cenocepacia* strains when grown in SCFM2 and LB media. In all three strains, phytomonic acid was detected at higher levels in SCFM2 cultures compared to LB. Plot was generated using EtOAc extracts at 24 and 48 h time points, as this method best extracted phytomonic acid.

Supplementary Table S1. Theoretical *m/z* for all annotated compounds identified in the MS/MS network.

Compound number	Compound Name	Theoretical *m/z*
**1**	Methylated pyochelin (enantio-thiazostatin)	339.084
**2**	HPTzTn-COOH	307.021
**3**	Aeruginoic acid	222.022
**4**	Aerugine	210.058
**5**	C8-AHL	228.159
**6**	C8-AHL (hydrolyzed)	246.170
**7**	C13-AHL (hydrolyzed)	316.248
**8**	C13:1db-AHL (hydrolyzed)	314.233
**9**	C13-AHL (hydrolyzed) [M+Na]	338.230
**10**	3-OH-C13-AHL (hydrolyzed) [M+Na]	354.225
**11**	Fragin	274.213
**12**	PNO B	181.134
**13**	PNO A	197.129
**14**	dPNO	199.144
**15**	2-Isopropyl-3-methoxypyrazine	153.102
**16**	2,5-Diisopropylpyrazine	165.139
**17**	Bacteriohopanetetrol cyclitol ether	708.541
**18**	Bacteriohop-6-enetetrol cyclitol ether	706.525
**19**	Bacteriohopanepentol cyclitol ether	724.536
**20**	Bacteriohop-6-enepentol cyclitol ether	722.520
**21**	Phytomonic acid (lactobacillic acid)	297.279
**22**	MG(19:1)	371.316
**23**	MG(18:1)	357.300
**24**	MG(17:1)	343.284
**25**	MG(16:1)	329.269
**26**	MG(17:1) [M+Na]	365.266
**27**	2-OH-PE(16:1)	468.272
**28**	2-OH-PE(16:0)	482.288
**29**	2-OH-PE(17:1)	510.319
**30**	2-OH-PE(18:1)	496.303
**31**	2-OH-PE(18:0)	498.319
**32**	2-OH-PE(19:1)	510.319

Key: PNO – pyrazine *N*-oxide, AHL – *N*-acylhomoserine lactone, MG – monoacylglycerol, PE – phosphatidylethanolamine.

Supplementary Table S2. List of trimethoprim metabolites observed in SCFM2 and LB media. Features were identified by MS2LDA to share a common motif (Mass2Motiff 543) corresponding to the trimethoprim substructure.

Theoretical *m/z*	SCFM2	LB
365.1448	+	-
368.1460	+	-
389.2509	+	-
391.1974	+	+
411.1320	+	-
457.1705	+	-
473.1492	+	-
489.1438	+	-
501.1441	+	-
503.1417	+	+
503.1554	+	-
505.1567	+	-
519.1373	+	+
521.1522	+	-
535.1674	+	+
537.1428	+	-
548.0357	+	-
549.1294	+	+
549.1450	+	-
551.1444	+	+
553.1432	+	+
564.1400	+	-
565.1245	+	+
567.1396	+	+
597.1327	+	-
609.1500	+	-
703.1411	+	-
735.2838	+	-
749.1256	+	-
